# Fast sampling of protein conformational dynamics

**DOI:** 10.1126/sciadv.aea4617

**Published:** 2026-03-27

**Authors:** Michael A. Sauer, Souvik Mondal, Brandon Neff, Sthitadhi Maiti, Matthias Heyden

**Affiliations:** School of Molecular Sciences, Arizona State University, Tempe, AZ 85287, USA.

## Abstract

Protein function often depends on dynamic transitions between conformations rather than just static structures. However, our current ability to characterize or predict such dynamics lags behind recent advances in protein structure prediction. Enhanced sampling methods can speed up molecular dynamics simulations to study protein conformational transitions but require prior knowledge of key collective motions involved. Here, we demonstrate for a series of proteins of varying complexity that the required information is encoded in anharmonic low-frequency vibrations. Using recently developed methods, we show that this information can be easily extracted from short dynamics simulations without requiring prior knowledge. Combined with enhanced sampling, we correctly predict conformational transitions in all test proteins and generate highly reproducible free energy landscapes. This allows for the rapid generation of accurate protein conformational ensembles, which is critical to unravel the complex relationship between protein sequence, structure, and dynamics.

## Teaser

Anharmonic low-frequency vibrations of proteins can be used to predict conformational ensembles and free energy surfaces.

## INTRODUCTION

Recent advances in protein structure prediction driven by machine learning [e.g., AlphaFold (*1*–*4*), ESMfold (*5*), and RoseTTAFold (*6*, *7*)] have markedly improved structure-based designs of enzymes and other proteins [e.g., RFdiffusion (*8*)]. However, protein function is often not the result of a single stable conformation but involves reversible conformational transitions between two or more distinct states, i.e., protein dynamics (*9*–*11*). Thus, de novo computational enzyme design is currently limited by our ability to describe the more complex relationship between protein sequence, structure, dynamics, and function. Recent developments, such as the Biomolecular Emulator (BioEmu), are already capable of generating realistic equilibrium conformational ensembles for proteins, but are limited to single protein chains in solution or the prediction of a single thermodynamic state (300 K) and are not yet able to describe the impact of ligand binding (*12*).

The characterization or prediction of protein dynamics requires information on associated free energy surfaces (FESs). Stability differences between distinct conformational states that reversibly interconvert are small by definition and thus challenging to predict reliably. Despite the existence of large molecular dynamics (MD) trajectory datasets as used during training of BioEmu (*12*), the availability of data on reliable protein conformational ensembles remains limited. Compared to hundreds of thousands of experimentally determined protein structures in the Protein Data Bank (PDB) (*13*), only a handful of proteins have been studied in sufficient detail, either experimentally or computationally, to provide reliable information on conformational dynamics.

With a few exceptions [e.g., time-resolved x-ray crystallography (*14*, *15*) and cryo–electron microscopy (*16*–*18*)], many experiments characterize protein conformational dynamics only in terms of distance fluctuations between individually labeled sites (*19*). Even sophisticated multicolor single-molecule fluorescence resonance energy transfer experiments (*20*–*23*) that track multiple distances simultaneously do not provide fine-grained information on conformational changes.

Classical MD simulations play a critical role in expanding the available data on protein conformational transitions at an atomistic level. Enhanced sampling simulations along suitable collective variables (CVs) can drastically accelerate the sampling of slow conformational dynamics (*24*, *25*). However, without prior knowledge of suitable CVs to drive conformational transitions in biased simulations, MD simulations of proteins are typically undersampled [with notable exceptions; (*11*, *26*–*29*)] and remain too costly to run on timescales associated with relevant biomolecular functions (often milliseconds to seconds).

Determining a suitable set of low-dimensional CVs to explore the dynamics of a given protein has remained a pervasive challenge. Selecting CVs typically requires prior knowledge of the conformational changes to be observed, and, ideally, CVs align with the most likely transition pathways. Identifying suitable CVs without prior knowledge is an active field of research (*30*–*35*).

We recently developed a theoretical framework that allows for the analysis of anharmonic low-frequency vibrations in molecules including proteins: frequency-selective anharmonic (FRESEAN) mode analysis (*36*). In contrast to low-frequency harmonic or quasiharmonic normal modes, which have historically been envisioned as potential CVs to explore protein conformational space (*37*–*40*), FRESEAN mode analysis does not rely on approximations that limit the validity of identified low-frequency vibrations. Instead, FRESEAN mode analysis can isolate collective degrees of freedom that describe structural distortions associated with minimal restraining forces (*36*). Simulations of a small test system (alanine dipeptide) indicate that such motions describe suitable CVs to enhance the sampling of conformational dynamics (*41*).

To our knowledge, we present the first enhanced sampling simulations based on anharmonic low-frequency vibrations of entire proteins. Specifically, we developed a modified approach to FRESEAN mode analysis to facilitate its application for systems with a large number of degrees of freedom. We then used this approach to extract CVs for enhanced sampling simulations for a test set of proteins with well-studied conformational dynamics.

In the following, we show that FRESEAN mode analysis of short MD simulations yields highly reproducible vibrational modes at low frequencies. Enhanced sampling simulations using these modes as CVs reliably sample known conformational transitions in each test protein. The approach does not require prior knowledge of expected conformational transitions and can be performed in a single day using standard high-performance computing hardware. This high-throughput generation of conformational ensembles enables the creation of larger and more diverse datasets required to train next-generation machine learning models capable of describing combined relationships between protein sequence, structure, and dynamics.

## RESULTS

### Anharmonic low-frequency modes in proteins

To test our hypothesis that anharmonic low-frequency vibrations describe natural CVs for enhanced sampling simulations of protein dynamics, we focused on five well-studied protein systems: hen egg-white lysozyme (HEWL) (*40*), HIV-1 protease (HIV-1 Pr) (*42*), myeloid cell leukemia 1 (MCL-1) (*43*), ribose-binding protein (RBP) (*44*), and the Kirsten rat sarcoma virus (KRAS) protein (*45*) (bound to guanosine diphosphate). This diverse set includes a homodimeric complex (HIV-1 Pr) and a multidomain protein (RBP) in addition to single-domain proteins (HEWL, MCL-1, and KRAS). The structure of one of the proteins (HEWL) is further stabilized by four internal disulfide bridges.

For each protein, we applied FRESEAN mode analysis (*36*) to extract anharmonic low-frequency modes from all-atom MD trajectories (see Methods and Supplementary Text). FRESEAN mode analysis is based on a time-correlation formalism of atomic velocity fluctuations that identifies, at any given frequency, collective vibrations by their contributions to the vibrational spectrum [i.e., the vibrational density of states (VDoS) defined in eq. S2] (*36*). A full set of orthogonal normal modes can be generated for any sampled frequency, but we focus our analysis here exclusively on modes generated at zero frequency. Harmonic normal modes are ill-suited to describe zero-frequency processes because they involve nonoscillatory, diffusive, or collective motions that do not obey the linear restoring-force framework of the harmonic approximation. Such restrictions do not apply to FRESEAN mode analysis, which assumes neither harmonic behavior nor a single set of orthogonal modes (*36*).

To reduce the computational cost of FRESEAN mode analysis for systems with many degrees of freedom, we recently introduced a coarse-grained representation (two beads per residue; see also Supplementary Text for details) of all-atom protein trajectories (*46*). As shown in fig. S2, this representation can be used as input for the analysis and fully preserves collective low-frequency vibrations.

### Reproducible detection of low-frequency modes

To use anharmonic low-frequency vibrations as reliable CVs in enhanced sampling simulations, they must be easily reproduced from independent sets of simulations. To test this, we applied FRESEAN mode analysis to five replicas of 20-ns equilibrium trajectories for each protein (each replica equilibrated with randomized velocities; see Supplementary Text for details).

Modes 1 to 6 (at zero frequency) describe translational and rotational diffusion of the entire protein (see fig. S3) and are excluded from CV selection. Modes with indices 7 and larger describe vibrations whose contributions to the zero-frequency VDoS decrease with increasing mode index. We analyzed contributions of individual modes to the vibrational spectrum at all frequencies by projecting fluctuations in our trajectories onto each mode (*36*). For each system, we plotted these contributions for modes 1 to 10 (including translations and rotations) in fig. S4. Notably, the spectra in fig. S4 are free from other spectral signatures, e.g., vibrations at higher frequencies. This highlights the fact that the selected modes truly isolate low-frequency vibrations in the simulated proteins [in contrast to harmonic or quasiharmonic modes; see (*36*)]. Zero-frequency contributions of low-frequency vibrations (modes 7 to 10) result from the low-frequency tail of a peak at 5 to 13 cm^−1^, depending on the system. This is characteristic for anharmonic low-frequency vibrations and informs our CV selection for enhanced sampling simulations. In the following, we focus our analysis on modes 7 to 9, i.e., the three vibrations with the largest zero-frequency contribution to the VDoS.

**Fig. 1.**
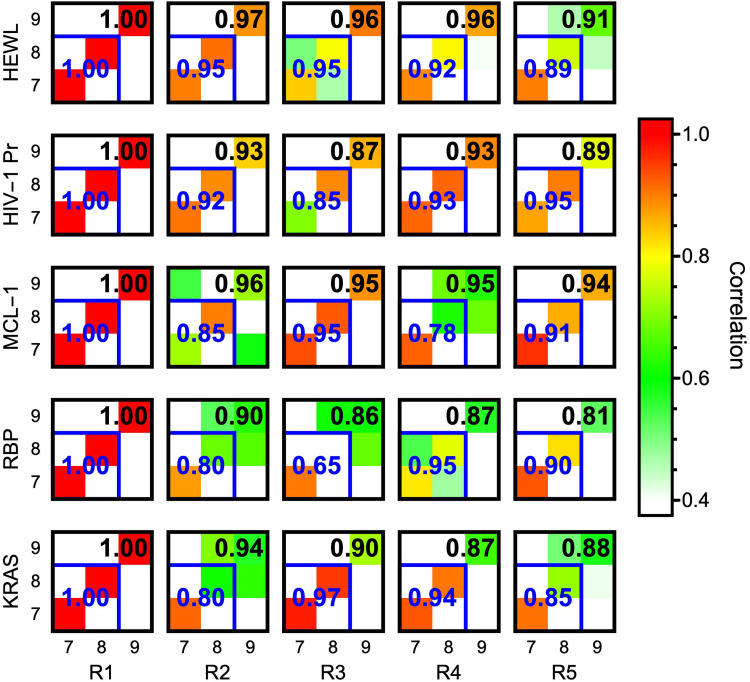
Reproducibility of zero-frequency FRESEAN mode analysis in five replica simulations. Matrices describing correlations between modes 7 and 9 in replicas R1 to R5 for each protein system (self-correlations with R1 are 1.0 by definition and only shown for clarity; see fig. S5 for all correlations). Colors of individual squares indicate the direct correlation between a pair of modes. Correlation coefficients between 2D (3D) subspaces described by modes 7 and 8 (7 to 9) in a pair of simulations are shown as blue (black) numerals.

A key question is whether our analysis provides reproducible assignments of anharmonic low-frequency modes. We investigate this in Fig. 1 using correlation coefficients between pairs of modes 7 to 9 from the five 20-ns replica simulations generated for each system (colored squares). For clarity, only correlations with replica R1 are shown (see fig. S5 for all correlations).

Correlations close to 1.0 (red) along the diagonal indicate that modes are reproduced in identical order. In some cases, correlation coefficients along the diagonal are smaller but accompanied by nonzero off-diagonal correlations. In these cases, it is more insightful to test whether the two-dimensional (2D) and 3D subspaces described by modes 7 and 8 and 7 to 9 are reproduced (see eqs. S13 and S14). These correlations are indicated in Fig. 1 as blue and black numerical values, respectively. Overall, the correlation analysis demonstrates that low-frequency vibrational modes are reproduced with high confidence between distinct replicas, despite the short length of the simulations (20 ns). The 2D subspaces in particular, spanned by modes 7 and 8 that we used as CVs for enhanced sampling simulations below, frequently exhibit high correlations. When the 2D subspaces for all replicas are compared with each other in every system, all but two pairs of replicas for RBP feature correlations >0.7. The same correlations are >0.9 in 6, 9, 4, 2, and 4 of 10 distinct replica pairs for HEWL, HIV-1 Pr, MCL-1, RBP, and KRAS, respectively. A common cause of reduced correlations between the 2D subspaces spanned by modes 7 and 8, e.g., for MCL-1, RBP, and KRAS, is mixing of modes 8 and 9 while mode 7 is reproduced with a higher correlation. This can be observed in fig. S5.

The high reproducibility of low-frequency vibrational modes from 20-ns simulations may initially seem unexpected. However, we note that even the lowest vibrational frequencies observed here occur at frequencies corresponding to one oscillation period every 3 ps (10 cm^−1^ ≈ 0.3 THz). Thus, each of our simulation trajectories averages over several 1000 oscillations.

Notably, this is not true for alternative approaches such as quasiharmonic or principal component modes that aim to describe molecular vibrations based on a single covariance matrix of atomic displacements. Correlations between pairs of low-frequency quasiharmonic modes obtained from the same trajectories, as well as 2D and 3D subspaces spanned by two or three modes, rarely reach 0.5 (figs. S6 and S7). Extending the length of simulations to the microsecond timescale improves the reproducibility of covariance matrix eigenvectors only moderately. This is shown in figs. S8 and S9 for principal component modes of HEWL computed from five 1-μs replica simulations. In contrast, the high reproducibility of low-frequency modes in Fig. 1 demonstrates that the time-correlation formalism used in FRESEAN mode analysis does not depend on rare events.

### Fast and consistent sampling of protein dynamics

**Fig. 2.**
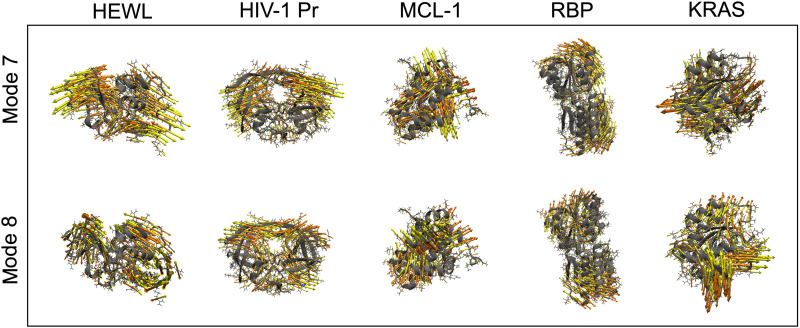
Visualization of anharmonic low-frequency vibrational modes used as CVs for enhanced sampling simulations. The displayed modes were selected for their contributions to the VDoS at zero frequency using FRESEAN mode analysis and are shown as displacement vectors (yellow and orange) relative to a reference structure.

Next, we tested the ability of anharmonic low-frequency modes to enhance the sampling of conformational dynamics. Specifically, we used modes 7 and 8, i.e., the two most prominent vibrations contributing to the VDoS at zero frequency, as CVs in enhanced sampling simulations with the well-tempered metadynamics protocol (*24*) (see Supplementary Text for details). For each protein, we visualized modes 7 and 8 as displacement vectors relative to a reference structure in Fig. 2 to illustrate the associated collective motions. We initially envisioned mode 7 as the main CV and mode 8 as an orthogonal auxiliary CV. However, both modes 7 and 8 contributed equally to the enhanced sampling for all systems studied here. We did not identify sampling issues that prompted us to add additional vibrational modes as CVs for enhanced sampling, but this is a potential option for future work.

**Fig. 3.**
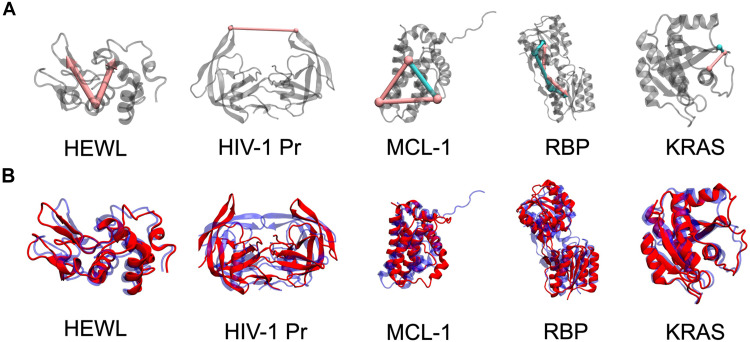
Geometric CVs and previously reported conformations for each studied system. (**A**) Visualization of distances and angles shown as colored cylinders with spherical end points within each protein structure (gray cartoons). Colors (cyan and magenta) are referenced in table S2. (**B**) Visualization of “closed” (blue) and “open” (red) states for each protein as secondary structure cartoons.

For each system, we performed metadynamics simulations using modes 7 and 8 as CVs that were obtained independently from the five replica simulations described above. Each simulation was stopped after 100 ns (less than 24 hours on a single Graphics Processing Unit or GPU) and analyzed for sampled conformational changes. To test whether these simulations provided consistent information on protein conformational dynamics, we first analyzed the biased simulation trajectories. Despite the presence of the biasing term, these trajectories provide insight into whether characteristic large amplitude motions are consistently sampled. We analyzed the latter using geometric variables (see table S2 and Fig. 3A) that have been introduced previously for each system (*40*, *42*, *43*–*45*). These geometric variables contain prior knowledge of the expected dynamics but are not part of our enhanced sampling protocol. They are only used to facilitate comparisons with previous work and to characterize the efficiency of our sampling scheme.

**Fig. 4.**
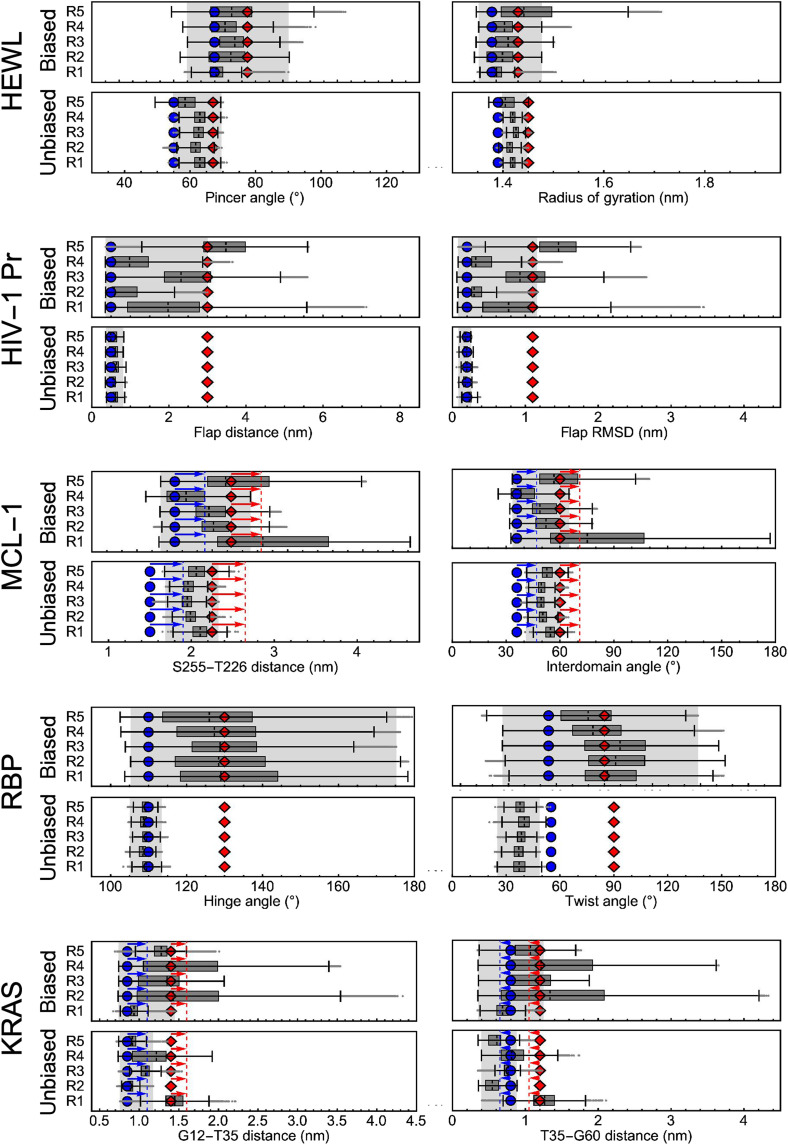
Box and whisker plots for each system illustrate the sampled conformational space in each metadynamics simulation replica. Plotted are distributions of two geometric variables, defined for each system in table S2 and visualized in Fig. 3A, computed directly from the biased trajectories along modes 7 and 8 and, for comparison, unbiased trajectories (see Supplementary Text for definition of box and whiskers). Blue circles and red diamonds indicate conformational states reported in the literature (for MCL-1 and KRAS, arrows and dashed lines indicate constant shifts derived from FESs in Fig. 5). Here, we generically refer to them as “closed” and “open” states and visualized them in Fig. 3B. Insets on the right illustrate representative superimposed structures from our simulations that correspond to “closed” and “open” states. RMSD, root mean square deviation.

The results are shown in Fig. 4 in direct comparison to unbiased simulations, where box and whisker plots show the extent of motion along the geometric variables. A gray background indicates the range of motion consistently described by each of the five independent replicas. Blue and red symbols indicate previously reported conformations for each system as visualized in Fig. 3B (for simplicity, we refer to them as “closed” and “open,” respectively; see Supplementary Text for details), and the insets display representative structures (*42*–*45*, *47*). Both conformations and transitions between them are consistently explored in each replica for HEWL, HIV-1 Pr, and RBP. For MCL-1 and KRAS, we observed constant shifts between coordinates reported in the literature (*43*, *45*) and free energy minima in our simulations (see next section). These shifts and the free energy minima observed in our simulations are indicated as arrows and vertical dashed lines, respectively, in Fig. 4. Taking this into account, we find that for MCL-1, four of five replicas sample both states, while one replica (R4) narrowly misses the shifted “open” state. For KRAS, we find that all five replicas sample the full range of Thr35-Gly60 distances associated with transitions between the “closed” to “open” states, while two replicas (R1 and R5) do not fully sample the Gly12-Thr35 distance assigned to the “open” state. Thus, considering all systems and five respective replicas, 22 of 25 metadynamics trajectories (88%) sample transitions between previously reported states in less than 100 ns (using CVs extracted from unbiased 20-ns simulations). We note that an extension of our metadynamics trajectories to 160 ns results in full sampling of “closed” to “open” transitions for every replica of each protein (see fig. S10).

The comparison to five replicas of unbiased 100-ns control simulations for each system in Fig. 4 demonstrates the impact of our enhanced sampling scheme. For all systems, enhanced sampling along anharmonic low-frequency modes increased the consistently sampled range of motion by at least twofold (closer to sixfold for HIV-1 Pr and RBP).

### Sampling FESs

FESs as a function of the respective CVs obtained directly from the five independent metadynamics simulations are shown in fig. S11. The independently sampled CVs used in each simulation are similar, as shown in Fig. 1, but not identical, which hinders direct comparisons between replicas. However, after unbiasing the metadynamics trajectories, we projected weighted conformational ensembles on the geometric variables introduced in Fig. 3 to construct FESs with a common set of variables (*48*). In contrast to the biased distributions shown in Fig. 4, the resulting FESs in fig. S12 allow us to distinguish high and low free energy conformations sampled during the metadynamics simulations. Correlations between geometric variables (sampled states fall on diagonal) indicate redundant information. We do not observe such correlations for the CVs used to enhance sampling in our simulations, i.e., modes 7 and 8 obtained from FRESEAN mode analysis at zero frequency, which are collective orthogonal motions by definition.

**Fig. 5.**
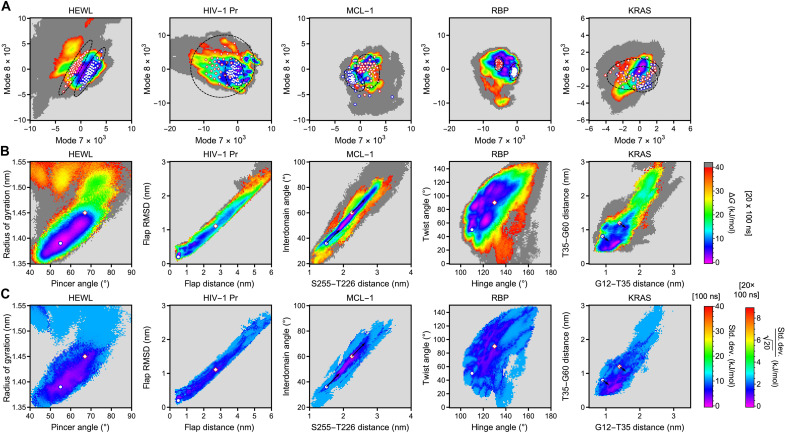
FESs and statistical errors obtained from 20 replica simulations for each system. (**A**) FESs as a function of CVs defined by anharmonic low-frequency vibrations. Open circles (blue) and diamonds (red) indicate 40 microstates of the “closed” and “open” ensembles (including constant shifts for MCL-1 and KRAS). Ellipses highlight the separation/overlap between both ensembles. (**B**) FESs as a function of geometric variables with the “closed” and “open” states indicated by open circles (blue) and diamonds (red). For MCL-1 and KRAS, arrows indicate constant shifts between prior reports of these states and free energy minima in our simulations. (**C**) Statistical uncertainties of the FESs in geometric variable space with “closed” and “open” states and shifts indicated as in (B).

Comparisons of FESs obtained for distinct replica simulations of the same protein (fig. S12) show that sampling remains insufficient to fully characterize the conformational ensembles. Therefore, we increased the number of replica metadynamics simulations for each system to 20 (starting structures sampled every 5 ns from unbiased 100-ns trajectories), each with 100 ns of metadynamics sampling and parallel processing. Here, we used identical vibrational modes as CVs (obtained for R1 above) in each replica to allow for direct comparisons and averaging of thermodynamic ensembles (see Supplementary Text for details). In Fig. 5, we plot the resulting FESs (obtained from averaged weighted probability distributions; see Supplementary Text) both in the space defined by vibrational modes (panel A) and geometric variables (panel B). Statistical errors, i.e., the standard deviation describing the uncertainty of a single 100-ns simulation and the standard error of the mean for all 20 replica simulations, are shown as a function of the geometric variables in panel C. These statistical errors indicate uncertainties of less than ±10 kJ/mol for a single 100-ns simulation and less than ±3 kJ/mol after averaging. This is further emphasized in fig. S13, in which we plot a 1D minimum free energy path for the “closed” to “open” transition in HEWL including statistical error bars (standard deviation and standard error of the mean). Such small uncertainties are remarkable given that error bars for protein FESs are often not even reported (*40*, *42*–*45*). The generation of converged FESs for five distinct protein systems, obtained with the same general protocol, highlights the role of anharmonic low-frequency vibrations as drivers of conformational change.

We indicate previously reported conformations (*42*–*45*, *47*) (referred to as “closed” and “open” in analogy to Fig. 4) with blue circles and red diamonds in Fig. 5 (B and C). Our simulations accurately reproduce previously reported conformations (apart from constant shifts for MCL-1 and KRAS), while revealing additional details on the FES that have so far not yet been described. We further report free energy differences between the “closed” and “open” states for all five systems in fig. S14. The success of low-frequency vibrations to enhance sampling of previously observed conformational changes is readily explained upon projection of the “closed” and “open” state ensembles into our CV space in panel A. Notably, any pair of geometric variables (a single point in Fig. 5B) describes an ensemble of conformations in alternative representations. In Fig. 5A, we indicate 40 conformations representative of the “closed” and “open” states for each system (selected via *k*-means clustering) in the CV spaces defined by low-frequency vibrations. With the exception of KRAS, the “closed” and “open” conformations are well separated in CV space (highlighted by ellipses), confirming that low-frequency vibrations used as CVs are directly related to the conformational dynamics reported previously in the literature (*40*, *42*–*44*). The overlap between “closed” and “open” states of KRAS in our CV space indicates that the low-frequency vibrations detected for KRAS describe independent collective motions that are not directly coupled to conformational dynamics reported previously for this system. Nevertheless, sampling is enhanced for KRAS due to indirect effects, e.g., the biased motion along vibrational modes allows the system to access alternative transition pathways with lower free energy barriers.

### Comparison to direct biasing with geometric variables

**Fig. 6.**
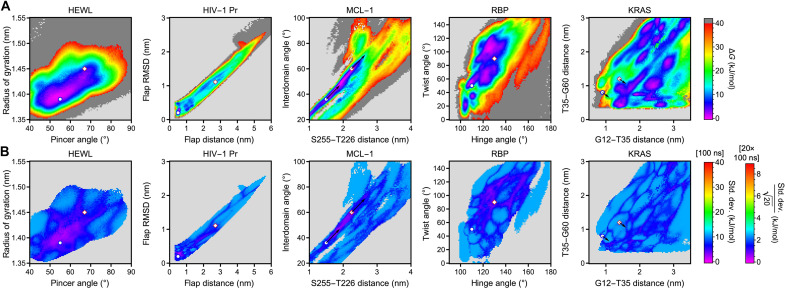
FESs and statistical errors obtained from 20 replica simulations for each system generated with biasing potentials applied directly along geometric variables. (**A**) FESs with the “closed” and “open” states indicated analogously to Fig. 5. (**B**) Statistical uncertainties of the FESs with “closed” and “open” states and shifts indicated as in (A).

To further evaluate the ability of CVs based on low-frequency vibrations to enhance sampling, we performed a second set of metadynamics simulations (20 × 100 ns for each system) in which the simulations were biased directly along the geometric variables introduced in Figs. 3 to 5. The results are shown in Fig. 6.

The similarities and differences between both types of enhanced sampling simulations in Figs. 5 and 6 are insightful. For HEWL, the FESs obtained in both cases are quite similar apart from differences in the shape of the free energy minimum, which are in line with statistical uncertainties shown in Figs. 5C and 6B. For HIV-1 Pr, free energy minima of “closed” and “open” states are seen in both simulations. However, sampling along low-frequency vibrations appears to follow a lower free energy path. We attribute the latter to the ability of low-frequency vibrations to identify paths of least resistance for conformational change, which has been the initial motivation of our approach. For MCL-1, the positions of the minima vary depending on the CVs used for biasing, which indicate that one or both sets of CVs result in incomplete sampling. Notably, simulations biased along the geometric variables sample partially unfolded high free energy states (Ser255-Thr226 distances of >3 nm). In contrast, simulations biased along low-frequency vibrations limit themselves to conformational dynamics within the native ensemble.

For RBP, the free energy minima of previously reported states are reasonably well reproduced with both sets of CVs. A third free energy minimum at a hinge angle of 120° and a twist angle of 60° is also reproduced in both cases. However, other features in this more complex FES show variations that again indicate incomplete sampling in one or both cases.

For KRAS, we observe the most drastic differences between simulations biased along low-frequency vibrations and geometric variables, in this case, two residue-residue distances. Specifically, the latter do not observe a stable “closed” state but identify multiple free energy minima with residue-residue distances that increase by several nanometers from the native structure and correspond to partially unfolded states (see fig. S15). This indicates substantial sampling problems, which we attribute to the noncollective character of the residue-residue distances used as CVs. In contrast, metadynamics simulations biased along intrinsically collective low-frequency vibrations in Fig. 5 sample primarily natively folded conformations and transitions between the previously reported “closed” and “open” states.

Except for KRAS, where biasing potentials applied to residue-residue distances primarily sample partially denatured states, it is nontrivial to determine which set of FESs contains more reliable information. To provide more insight, we measure the information content of each FES in both CV spaces via the Shannon entropy, *S*_2_ (measured in bits as defined in eq. S24). For each CV space, we define the *S*_2_ of the FES obtained from simulations biased directly in that space as a reference (100%) and compare it to the *S*_2_ of an FES obtained after reweighting simulations biased along a distinct set of CVs. We apply the logic that a reliable set of CVs should lose less information upon reweighting from one space into another, while a less reliable set of CVs loses more information. The results are shown in Table 1 below, in combination with Bhattacharyya coefficients (BCs; defined in eq. S25) that describe the similarity of the FES pairs, i.e., the corresponding probability distributions. For HEWL and HIV-1 Pr, sampling in vibrational mode space and reweighting into the space of geometric variables recover a larger fraction of *S*_2_ (94 and >100%, respectively) compared to the inverse case, i.e., sampling in geometric variable space and reweighting into the vibrational mode space (85 and 77%, respectively). For MCL-1 and RBP, reweighting from one space into the other is essentially symmetric. For MCL-1, 87% of the reference entropy is recovered when reweighting from one space into the other in both cases. For RBP, no information is lost at all, indicating that both sets of CVs are essentially equivalent.

**Table 1.** BCs and *S*_2_ (in bits) of probability distributions defined by FESs sampled for each system using zero-frequency modes 7 and 8 or geometric variables as CVs to apply bias potentials in metadynamics simulations. We use BCs to compare pairs of probability distributions and *S*_2_ to measure their information content in a specific CV space. In each CV space, the *S*_2_ obtained from biased simulations sampled directly in that space is defined as a reference (100%).

	CV space	Modes 7 and 8		Geometric var.	
	Biasing	Modes 7 and 8	Geometric var.	Modes 7 and 8	Geometric var.
HEWL	BC	0.76		0.89	
	*S*_2_	7.42	6.27	6.25	6.63
		(100%)	(85%)	(94%)	(100%)
HIV-1 Pr	BC	0.63		0.84	
	*S*_2_	9.23	7.10	8.05	7.56
		(100%)	(77%)	(106%)	(100%)
MCL-1	BC	0.78		0.77	
	*S*_2_	8.52	7.42	6.08	6.98
		(100%)	(87%)	(87%)	(100%)
RBP	BC	0.81		0.84	
	*S*_2_	9.92	9.92	8.83	8.77
		(100%)	(100%)	(101%)	(100%)
KRAS	BC	0.77		0.17	
	*S*_2_	7.97	9.56	6.92	9.66
		(100%)	(120%)	(72%)	(100%)

Results for KRAS are shown for completeness: The population of partially unfolded states when biasing potentials are applied to residue-residue distances results in a high entropy in both CV spaces. However, this information is not relevant for conformational transitions within the folded ensemble.

While the results in Table 1 do not allow us to decide unambiguously which FES is more reliable for each system, it does show that CVs based on low-frequency vibrations preserve, on average, more information during reweighting of biased trajectories than geometric variables proposed in the literature. At the same time, biasing along low-frequency vibrations appears to have a higher chance to preferentially sample conformational transitions within the folded state. This avoids partial unfolding, which is often irreversible even in enhanced sampling simulations (*49*). This adds to the advantage that no system-specific knowledge is required to determine the low-frequency vibrations using FRESEAN mode analysis.

## DISCUSSION

We introduced anharmonic low-frequency vibrations as natural CVs for enhanced sampling simulations of protein conformational transitions. Such vibrations can be extracted reproducibly from short unbiased simulation trajectories on the nanosecond timescale using a FRESEAN mode analysis (*36*). Our approach enables enhanced sampling simulations of protein conformational dynamics without requiring prior knowledge of the type of motions involved.

We follow longstanding conceptual ideas on the relationship between low-frequency vibrations, barrier crossing events, and conformational transitions (*37*–*39*, *50*, *51*). The key advance here is the possibility to isolate true low-frequency vibrations without simplifying the system or implying harmonic approximations that are invalid in the low-frequency limit. The absence of high-frequency fluctuations (see fig. S4) along the identified modes ensures that only minimal biasing forces are sufficient to alter the protein conformation. Consequently, biasing simulations along the identified modes consistently speeds up protein conformational sampling.

Averaging over multiple parallel simulations on timescales compatible with processing times of less than 24 hours on GPUs for many systems can produce converged FESs and corresponding conformational ensembles. Such high throughput enables the generation of large datasets and systematic studies of individual proteins, e.g., by comparing the dynamics of distinct mutants. Fast and efficient strategies to sample protein conformational ensembles are critical for the development of predictive models that combine protein structure and dynamics.

Mutations can modify the conformational ensemble of a protein in distinct ways, either through a change of low-frequency vibrations and related conformational transition pathways or via changes in the relative stability of distinct conformations. We note that for the systems studied here, we were able to extract low-frequency vibrations of the protein in one state to enhance the sampling of conformational transitions into another state. More complex systems, such as larger biomolecular complexes, may involve a sequence of conformational transitions that do not follow the same pathway. In such cases, we anticipate an iterative approach, in which low-frequency vibrations are resampled in each newly discovered conformation.

We do expect limitations of our approach for systems that involve substantial disorder. Intrinsically disordered regions (IDRs) in proteins are likely to give rise to a large number of low-frequency vibrations, which impedes the selection of a small number of vibrations as CVs for enhanced sampling. In such cases, the vibrational analysis can be restricted to folded regions of the protein, and interactions with IDRs are treated as part of the environment, e.g., the solvent.

State-of-the-art predictors for stable protein structures (*1*, *2*, *4*–*8*) were enabled by the existence of extensive datasets ranging from known protein structures to vast numbers of genomic sequences (*13*, *52*). The first successful machine learning–based predictions of protein conformational ensembles have been achieved for intrinsically disordered proteins, using datasets generated with computationally efficient coarse-grained polymer simulations (*53*–*55*). Recent developments such as BioEmu are already providing the framework for the training of the next generation of machine learning models capable of predicting the conformational ensembles of folded proteins (*12*). However, larger and more diverse datasets of conformational ensembles are needed to overcome remaining limitations. The enhanced sampling scheme presented here, which can be applied without prior knowledge of expected conformational transitions, provides an efficient approach to generate such datasets.

## MATERIALS AND METHODS

Simulation protocols for all-atom MD simulations of all systems are described in Supplementary Text, combined with a description of their coarse-grained representations, details on the FRESEAN mode analysis, and the well-tempered metadynamics simulations. Briefly, all simulations were performed with GROMACS 2022.5 and started from crystal structures reported in PDB entries 1HEL for HEWL, 1BVE for HIV-1 Pr, 3WIX for MCL-1, 1DRJ for RBP, and 5WCC for KRAS. Force fields used for each system were selected in accordance with prior work in the literature to allow for direct comparisons. Each protein was protonated assuming neutral pH conditions and solvated in a cubic box in aqueous solution with 150 mM NaCl (see table S1 for details).

With position restraints applied to nonhydrogen protein atoms, the energy of each system was minimized, and five replicas of each system were equilibrated in the isobaric-isothermal (NPT) ensemble with independently sampled initial velocities. For each replica of each system, we then performed unrestrained NPT simulations for 20 ns to perform the FRESEAN mode analysis. For the analysis, protein trajectories (coordinates and velocities stored every 20 fs) were (i) rotated into a reference coordinate system based on the crystal structure and (ii) coarse-grained into beads (one bead for glycines and two beads for each other amino acid). These simulation parameters were selected based on results from prior work (*46*). A matrix of mass-weighted time velocity cross-correlation functions was computed for all coarse-grained degrees of freedom with a maximum correlation time of 2 ps. We time-symmetrized each matrix term and performed a Fourier transform into the frequency domain with a 10 cm^−1^ Gaussian window.

The eigenvectors (modes) of the zero-frequency matrix describe displacement vectors corresponding to translational motion (modes 1 to 3), rotational motion (modes 4 to 6), and anharmonic low-frequency vibrations (modes 7+ with nonzero eigenvalues). We used modes 7 and 8 to define CVs for 100-ns well-tempered metadynamics simulations with the PLUMED-2.8.2 plugin for GROMACS. The CVs are evaluated by (i) aligning the protein to the equilibrated starting structure as a reference, (ii) switching to coarse-grained representation, (iii) computing displacement vectors relative to the reference structure, and (iv) projecting the displacement vectors onto the mode vector. Metadynamics simulations were performed for each replica of each system (five systems × five replicas × 100 ns).

In addition, we performed for replica R1 of each system an additional 100-ns unbiased simulation from which we extracted 20 sets of coordinates and velocities every 5 ns. These states were then used to start an additional set of 20 metadynamics simulations for each system (five systems × 20 simulations × 100 ns) to analyze the convergence of FESs.

## Supplementary Materials

### This PDF file includes:

Supplementary Text Figs. S1 to S15 Tables S1 and S2 References
