# Supplementary material for: Fast sampling of protein conformational dynamics
Source: Sci Adv. 2026 Mar 27;12(13):eaea4617. doi: 10.1126/sciadv.aea4617 (PMC13025022; doi:10.1126/sciadv.aea4617)
Supplement: Supplementary file 1 — Supplementary Text Figs. S1 to S15 Tables S1 and S2 References [file sciadv.aea4617_sm.pdf]

Science Advances
AAAS

Supplementary Materials for

Fast sampling of protein conformational dynamics

Michael A. Sauer *et al.*

Corresponding author: Matthias Heyden, mheyden1@asu.edu

Sci. Adv. **12**, eaea4617 (2026)
DOI: 10.1126/sciadv.aea4617

This PDF file includes:

Supplementary Text
Figs. S1 to S15
Tables S1 and S2
References

Supplementary Text

Simulation protocol

All simulations were performed using the GROMACS 2022.5 software package (*58*). Simulations were performed on five systems, including Hen Egg White Lysozyme (HEWL; 1HEL.pdb), HIV-1 Protease (HIV-1 PR; 1HHP.pdb), Myeloid cell leukemia-1 (MCL-1; 3WIX.pdb), K-Ras (5W22.pdb) and Ribose-Binding Protein (RBP; 1DRJ.pdb, G134R mutant). Molecular mechanics force fields used and the number of water molecules and ions included in each system are listed in table S1 and follow previous simulation studies of these systems in the literature (*42–45, 47*). All simulations were performed with the TIP3P water model (*59*) for Amber-family force fields and the modified TIP3P model (also known as TIPS3P) for CHARMM-family force fields (*60*). In accordance to previous literature, RBP, more precisely its G134R mutant, carried no charge and was simulated in pure water (*44*).

Protein Name	PDB	Force Field	Water	Na^+	Cl^-
Hen Egg White Lysozyme (HEWL)	1HEL.pdb	Amber99sb (*61*)	25644	73	81
HIV-1 Protease (HIV-1 Pr)	1HHP.pdb	Amber14sb (*62*)	25342	73	77
Myeloid Cell Leukemia-1 (MCL-1)	3WIX.pdb	CHARMM36m (*63*)	16056	46	47
K-Ras (KRAS)	5W22.pdb	Amber14sb (*62*)	22933	68	61
Ribose Binding Protein (RBP)	1DRJ.pdb	CHARMM36m (*63*)	21977	0	0

Table S1: Simulation Parameters for Studied Systems

First, the energy of each system was minimized using the steepest descent algorithm for 1000 steps. Then, the system was equilibrated in the isobaric-isothermal (NPT) ensemble at 300 K and 1 bar for 100 ps. For the equilibration simulations, we used a 1 fs integration time step, a velocity rescaling thermostat (*64*) with a 1.0 ps time constant, and a stochastic cell rescaling barostat (*65*) with a time constant of 2.0 ps.

This was followed by an unbiased 20 ns simulation in the NPT ensemble. In this simulation, we used a 2 fs integration timestep, a Nosé-Hoover thermostat (*66,67*) with a 1.0 ps time constant, and a Parrinello-Rahman barostat (*68*) with a 2.0 ps time constant. All covalent bonds involving hydrogens were constrained using the LINCS algorithm (*69*). Short-ranged electrostatic and Lennard-Jones

interactions were treated with a 10 Å real-space cutoff with energy and pressure corrections for dispersion interactions. Long-ranged electrostatic interactions were treated with the Particle Mesh Ewald algorithm (*70*) using a 1.2 Å grid. Coordinates and velocities were stored every 20 fs for subsequent analysis.

This simulation protocol, starting with the equilibration of the energy minimized structure, was repeated five times for each system using resampled starting velocities from a Maxwell distribution with a unique random seed. We then performed FREquency-SElective ANharmonic (FRESEAN) mode analysis (*36*) for each 20 ns trajectory to assess the reproducibility of the analysis protocol.

Well-tempered metadynamics simulations

Well-tempered metadynamics (WT-metadyn) (*24*) simulations were performed for each independent replica using the PLUMED 2.8 software package (*71, 72*). FRESEAN modes 7 and 8 at 0 THz were used as collective variables (CVs). Details on the coarse-grained representation of our CVs and the conversion to an all-atom representation for use in WT-metadyn simulations are described in the following sections. WT-metadyn simulations were run for 100 ns in the NPT ensemble with a 2 fs timestep. All other parameters (thermostat, barostat, bond constraints, short- and long-ranged interactions) were treated in the same way as for the unbiased 20 ns NPT simulations. Gaussian functions were added to the biasing potential every 1 ps with an initial height of 0.1 kJ/mol and a standard deviation of 0.001. The unit-less bias factor in the PLUMED implementation of metadynamics was set to 10. The free energy surface is recovered by inverting the sum of all Gaussian hills deposited throughout the simulation.

Coarse-grained representation of proteins

We defined the following coarse-grained representation of protein coordinates and velocities, which describes each amino acid (except glycine) with two beads: one bead represents the center of mass (COM) of all backbone atoms and one bead represents the COM of all side chain atoms (see figure S1). Glycine is treated as a single bead.

To perform WT-metadyn simulations along coarse-grained FRESEAN modes as CVs (modes 7 and 8 at zero frequency), we need to translate between the coarse-grained representation of FRESEAN modes (used in our analysis of unbiased simulations) and the all-atom representation

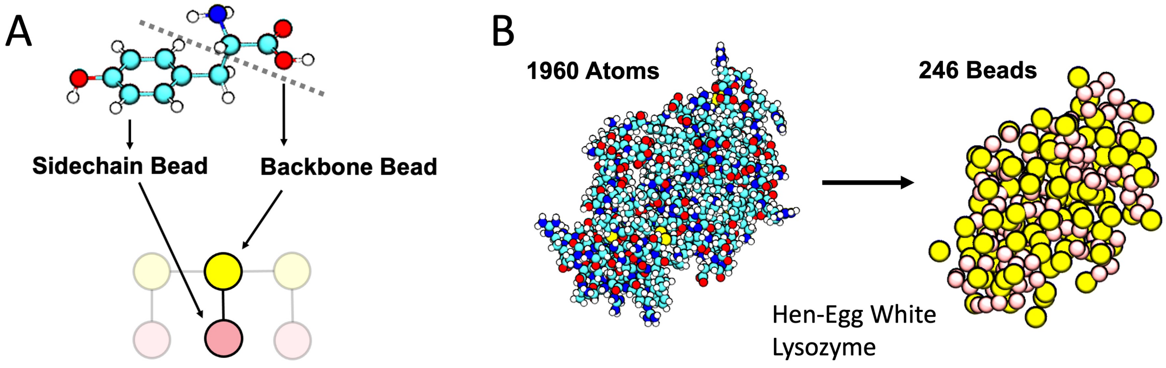

Figure S1: **Visualization of the two-bead coarse grained representation of amino acids.** (A) An example of the coarse-graining scheme for tyrosine, which shows which atoms are associated with either the backbone or side chain. In the coarse-grained representation, the corresponding backbone (yellow) and side chain (pink) beads correspond to the COM of the corresponding group of atoms. (B) Application of the coarse-graining scheme to HEWL. The coarse-graining scheme reduces the number of particles (all-atom: 1960 atoms; coarse-grained: 246 beads) and the dimensions of the velocity cross correlation matrices computed during FRESEAN mode analysis (see Ref. 36 and later section for additional information).

used in the WT-metadyn simulations. One option is to: A) evaluate the COM position for each group of atoms that correspond to a given bead during each step of the simulation; B) align the resulting coarse-grained structure to a coarse-grained reference structure; C) evaluate bead displacements relative to the reference; D) project a combined vector of all bead displacements on the coarse-grained FRESEAN modes used as CVs.

Here, we used an equivalent approach that is more straightforward to implement in PLUMED by converting the coarse-grained FRESEAN modes into a corresponding all-atom representation prior to the simulation. In this representation, per-bead components of a coarse-grained FRESEAN mode are distributed over the atoms contributing to each bead so that the sum of atomic components equals the corresponding per-bead components. During each step of the simulation, we then: A) align the all-atom protein structure to an all-atom reference structure; B) calculate atomic displacements relative to the reference; C) project a combined vector of all atomic displacements on the all-atom representations of FRESEAN modes used as CVs.

All-atom *vs.* coarse-grained vibrational density of states

To quantify whether coarse-grained protein trajectories reproduce low-frequency vibrations compared to an all-atom representation, we evaluated the vibrational density of states (VDoS) using both representations for an unbiased 20 ns HEWL trajectory. To resolve high-frequency vibrations prominently observed in the all-atom representation, we stored coordinates and velocities every 4 fs for this analysis.

For both representations, we define weighted velocities of either atoms or beads:

$$\tilde{\mathbf{v}}_i = \sqrt{m_i} \cdot \mathbf{v}_i \tag{S1}$$

Here, $\mathbf{v}_i$ is the velocity vector of atom i in the all-atom representation, or the velocity vector of bead i in the coarse-grained representation, *i.e.*, the COM velocity of its contributing atoms; m_i is either the mass of atom i in the all-atom representation or the mass of bead i in the coarse-grained representation, *i.e.*, the sum of its contributing atomic masses. We can define the VDoS of the protein via the Fourier transform of the sum over N (= number of atoms or beads) time auto correlation functions of weighted velocities $\tilde{\mathbf{v}}_i$.

$$\mathrm{VDoS}(\omega) = \frac{1}{2\pi} \cdot \frac{2}{k_B T} \int_{-\infty}^{+\infty} \exp(\mathrm{i}\omega\tau) \sum_i^N \langle \tilde{\mathbf{v}}_i(t) \cdot \tilde{\mathbf{v}}_i(t+\tau) \rangle_t \, d\tau \tag{S2}$$

Here, $\langle ... \rangle_t$ indicates the ensemble average over the simulation time. With this definition, the integral of the VDoS over positive frequencies corresponds to the number of degrees of freedom described by the velocities $\mathbf{v}_i$.

$$N_{\mathrm{DOF}} = \int_0^{+\infty} \mathrm{VDoS}(\omega) \, d\omega \tag{S3}$$

HEWL has 1960 atoms and 959 constraints (bonds involving hydrogens), which results in $N_{\mathrm{DOF}}^{\mathrm{aa}} = 3 \times 1960 - 959 = 4921$ in its all-atom representation. In the coarse-grained representation with 246 beads as shown in figure S1, the integral of the VDoS reduces to $N_{\mathrm{DOF}}^{\mathrm{cg}} = 3 \times 246 - 4.4 = 733.6$. Here, 4.4 is the number of effective constraints that remain in the coarse-grained representation (determined from the kinetic energy of the coarse-grained beads, the system temperature, and the equipartition theorem). The ratio $N_{\mathrm{DOF}}^{\mathrm{cg}}/N_{\mathrm{DOF}}^{\mathrm{aa}}$ describes how much information is lost overall due to coarse-graining. The ratio $\mathrm{VDoS}^{\mathrm{cg}}(\omega)/\mathrm{VDoS}^{\mathrm{aa}}(\omega)$ quantifies this information loss as a function of frequency. We plotted both, the all-atom and coarse-grained VDoS, in panel A of Figure S2 for frequencies, and their ratio in panel B.

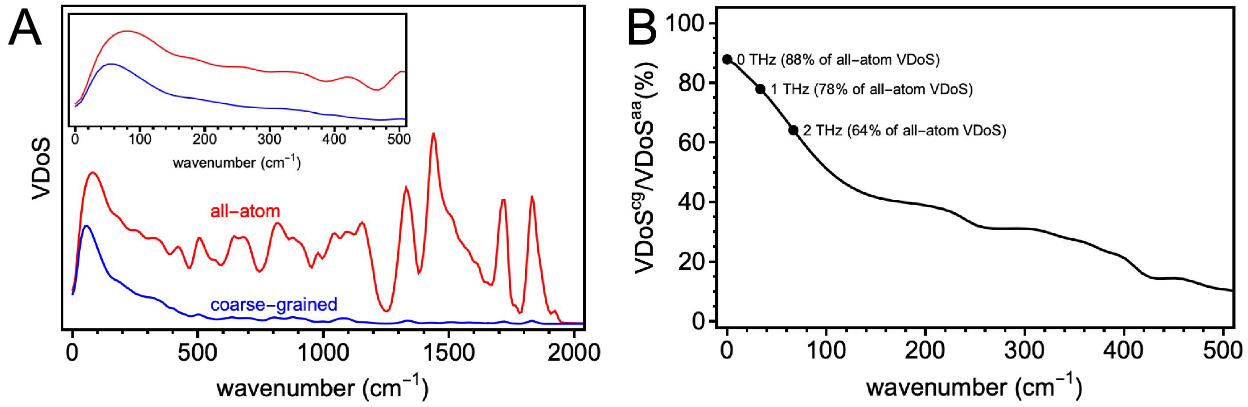

Figure S2: **Low-frequency vibrational spectrum in all-atom and coarse-grained representations.** (A) Vibrational Density of States (VDoS) calculated for the all-atom and coarse-grained representations of a 20 ns simulation of Hen Egg White Lysozyme (HEWL). The inset highlights frequencies <500 cm^{-1}. (B) Percentage of the all-atom VDoS information captured in the coarse-grained representation described by the ratio $\mathrm{VDoS}^{\mathrm{cg}}(\omega)/\mathrm{VDoS}^{\mathrm{aa}}(\omega)$.

The all-atom VDoS describes all vibrations in the protein including bond vibrations etc. within the backbone and side chains that are most prominent in the vibrational fingerprint region. These vibrations have no impact on the COMs used for our coarse-grained representation. Thus the coarse-grained VDoS is primarily missing vibrational degrees of freedom at high frequencies while low-frequency vibrations corresponding to relative vibrations of secondary structure units, side chain motions etc. are largely preserved. Correspondingly, we find that 88% of the zero frequency motions are captured in the coarse-grained representation, while the information loss sets in gradually with increasing frequency.

Since, our approach to identify CVs to enhance conformational sampling is based solely on zero frequency modes, figure S2 indicates that the coarse-grained protein representation contains effectively all information needed for FRESEAN mode analysis at zero frequency.

FREquency-SElective ANharmonic (FRESEAN) mode analysis

FRESEAN mode analysis is described in detail in our previous work in Ref. 36. We formulate the velocity cross correlation matrix with scaled velocities as in eq. S1. For simplicity, we use the index i to refer to a single degree of freedom, *i.e.*, the x-, y-, or z-component of a coarse-grained bead

velocity vector.

$$\tilde{v}_i = \sqrt{m} \cdot v_i \tag{S4}$$

While analyzing a simulation trajectory, we rotate coordinates and velocities of the protein trajectory into a common reference frame and compute the time cross-correlation matrix between mass-weighted velocities for all degrees of freedom i and j. We note that we only rotate into a reference coordinate system but do not subtract either translational or rotational velocities.

$$C_{\tilde{v},ij}(\tau) = \langle \tilde{v}_i(t)\tilde{v}_j(t+\tau)\rangle_t \tag{S5}$$

The Fourier transformation of each matrix element results in a frequency-dependent cross-correlation matrix with the following elements.

$$C_{\tilde{v},ij}(\omega) = \frac{1}{2\pi}\int_{-\infty}^{+\infty} \exp(\mathrm{i}\omega\tau) C_{\tilde{v},ij}(\tau) d\tau \tag{S6}$$

Notably, the trace of this matrix describes the Fourier transform of the sum of time auto correlations, which is identical to the vibrational density of states (VDoS) defined in eq. S2.

$$\mathrm{VDoS}(\omega) = \frac{2}{k_B T}\sum_i^{3N} C_{\tilde{v},ii}(\omega) \tag{S7}$$

This does not change if we diagonalize the matrix $\mathbf{C}_{\tilde{v}}(\omega)$ (with elements $C_{\tilde{v},ij}$) at each sampled frequency because the corresponding coordinate transformations are unitary. Therefore, we obtain an equivalent expression for the VDoS as the sum of eigenvalues λ_i at each frequency.

$$\mathrm{VDoS}(\omega) = \frac{2}{k_B T}\sum_i^{3N} \lambda_i(\omega) \tag{S8}$$

Consequently, at any specific frequency ω', the eigenvalues $\lambda_i(\omega')$ describe contributions of mass-weighted velocity fluctuations along the corresponding eigenvectors $\mathbf{Q}_i(\omega')$ to $\mathrm{VDoS}(\omega')$. An important observation is that the majority of eigenvalues $\lambda_i(\omega')$ are zero. Sorting the eigenvalue/eigenvector-pairs by the magnitude of $\lambda_i(\omega')$ (large to small) thus unambiguously identifies the collective degrees of freedom, *i.e.*, eigenvectors describing displacements relative to a reference structure, that contribute to the VDoS at frequency ω'.

At zero frequency, it is not surprising that eigenvalues $\lambda_{1-3}(\omega = 0)$, *i.e.*, contributions to $\mathrm{VDoS}(\omega = 0)$, are related to eigenvectors describing translations of the entire protein. Likewise,

eigenvectors associated with eigenvalues $\lambda_{4-6}(\omega = 0)$ describe essentially rigid body rotations. For HIV-1 Protease and RBP, we further observe some decoupling of the translational/rotational motions of the two distinct monomers and domains, respectively. We illustrated these eigenvectors, *i.e.*, zero-frequency modes 1-6, as displacement vectors for each coarse-grained bead superimposed with the visualization of an all-atom reference structure in figure S3. The diffusive character of the underlying motions is apparent from the analysis in the next section.

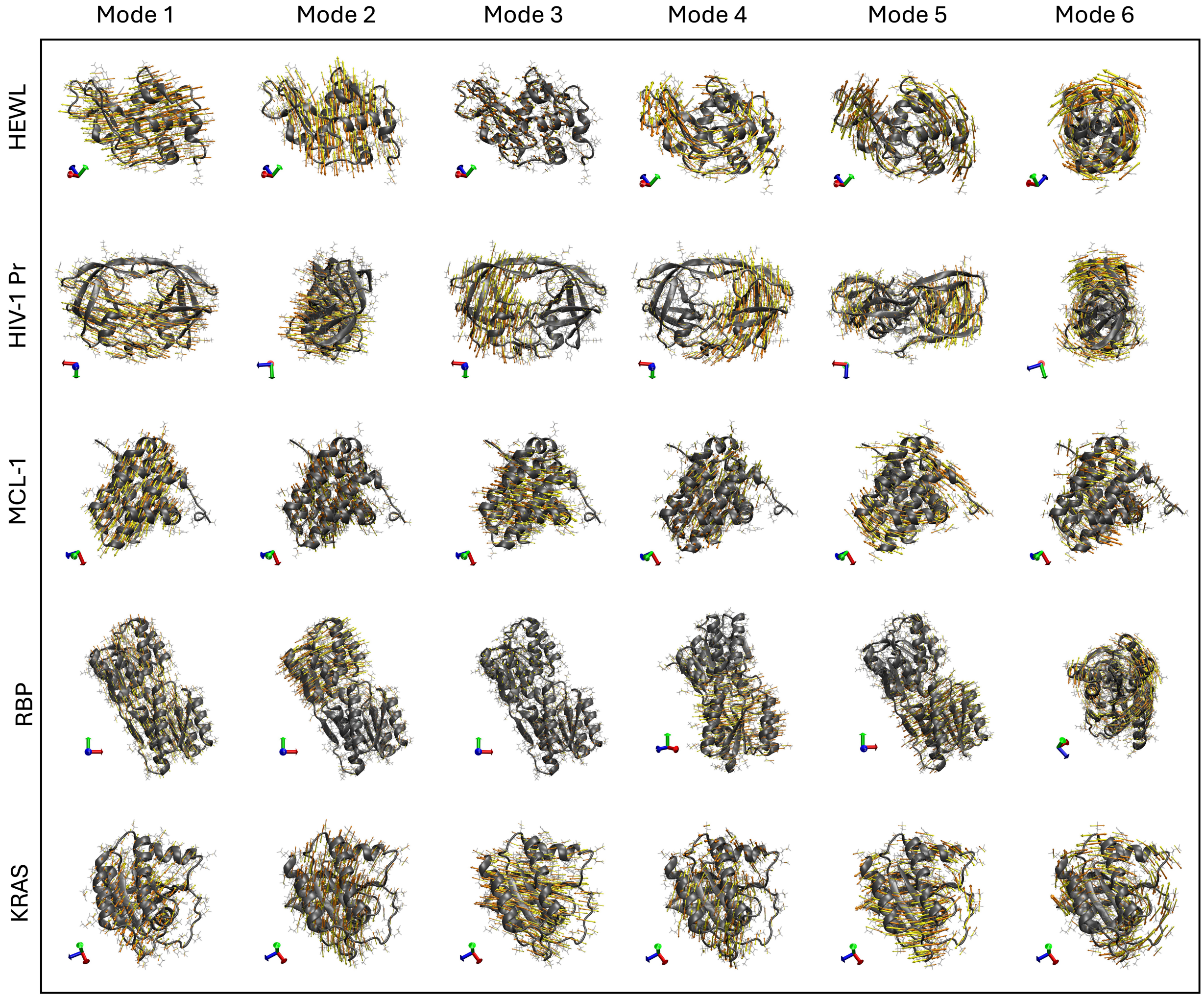

Figure S3: **Translational and rotational degrees of freedom.** Visualization of FRESEAN modes 1-6 (yellow/orange arrows) at zero frequency for the five studied systems. Modes 1-3 describe protein translations, while modes 4-6 describe rigid body rotations. Arrows in the bottom left of each rendering denote the protein orientation selected separately for clarity.

1D-VDoS for eigenvector projections

To illustrate that eigenvectors generated at zero frequency with FRESEAN mode analysis isolate diffusive motion (modes 1-6) and low-frequency vibrations (modes 7+), we computed the VDoS separately for the collective one-dimensional degrees of freedom described by the corresponding eigenvectors $\mathbf{Q}_i$ $[= \mathbf{Q}_i(\omega = 0)]$ for i from 1 to 10.

To do so, we first project weighted velocities ($\tilde{\mathrm{v}}(t)$) from our simulation trajectories on a given eigenvector $\mathbf{Q}_i$ with its $3N$ components q_i^j.

$$\dot{q}_i(t) = \sum_j q_i^j \tilde{\mathrm{v}}_j(t) \tag{S9}$$

This allows us to define a mass-weighted time correlation function for fluctuations along $\mathbf{Q}_i$, which we Fourier transform to obtain the VDoS for this single collective degree of freedom.

$$\mathrm{VDoS}_{Q_i}(\omega) = \frac{2}{k_B T}\left[\frac{1}{2\pi}\int_{-\infty}^{\infty} \exp\left(\mathrm{i}\omega\tau\right)\, \langle \dot{q}_i(t)\dot{q}_i(t+\tau)\rangle_t \, d\tau\right] \tag{S10}$$

We note that an equivalent result can be extracted directly from the frequency-dependent cross-correlation matrix $\mathbf{C}_{\tilde{\mathrm{v}}}(\omega)$.

$$\mathrm{VDoS}_{Q_i}(\omega) = \frac{2}{k_B T}\mathbf{Q}_i^{\mathrm{T}}\mathbf{C}_{\tilde{\mathrm{v}}}(\omega)\mathbf{Q}_i \tag{S11}$$

We plotted the resulting one-dimensional VDoS for modes 1-10 of each system in figure S4. The diffusive motion along modes 1-6 is evident through the peak position at 0 cm^{-1}, whose amplitude is proportional to the corresponding diffusion coefficient. For modes 7-10, we observe low-frequency vibrations with peak intensities between 5 and 13 cm^{-1} that contribute to the zero frequency VDoS via a low-frequency tail. We note that additional peaks are absent, highlighting the succesful isolation of low-frequency vibrations, in contrast to similar projections on low-frequency vibrational modes obtained with conventional methods that rely on harmonic approximations (see Ref. 36).

Reproducibility of FRESEAN mode analysis

A key feature of FRESEAN mode analysis is not only its ability to isolate low-frequency as shown in the previous section and Ref. 36, but a high reproducibility of low-frequency eigenvectors obtained from distinct simulations.

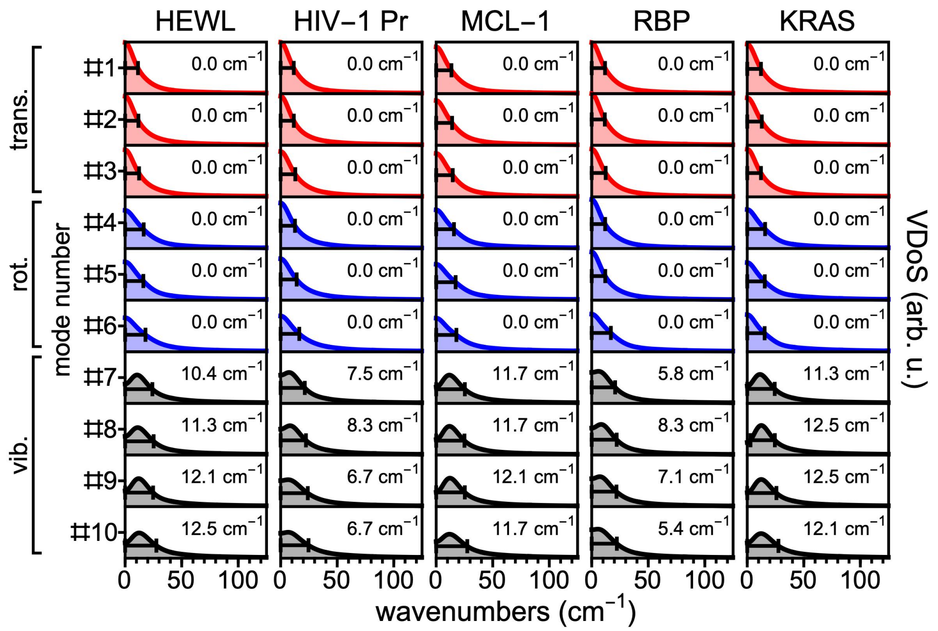

Figure S4: **VDoS of fluctuations along selected modes.** Visualization of the 1D-VDoS for individual FRESEAN modes 1-10 obtained at zero frequencies for all five systems. The 1D-VDoS for translational, rotational and low-frequency vibrational modes are shown in red, blue and black, respectively. Numbers indicate the peak position and a horizontal bar indicates the full width at half maximum.

To compare vibrational modes obtained from separate simulation trajectories, we compute the correlation coefficients between the corresponding normalized eigenvectors as a scalar product.

$$C_{(i,j)}^{(k,l)} = \left| Q_i^{(k)} \cdot Q_j^{(l)} \right| \tag{S12}$$

Here, the indices i and j indicate the eigenvector index and the indices k and l indicate the index of the simulation replica. Correlations between modes 7-9 in replica 1 and modes 7-9 in replica R1 to R5 are shown in figure 1 of the main text. In figure S5, for completeness, we show the corresponding correlations for all combinations of modes and simulation replicas.

In addition to comparisons between pairs of modes it is often useful to compare the sub-spaces described by a small set of modes obtained from two distinct simulations. This is indicated for the two-dimensional sub-spaces spanned by modes 7 and 8 and the three-dimensional sub-spaces spanned by modes 7, 8, and 9 as numerical values in figure 1 of the main text. To define the correlation between sub-spaces, we project eigenvectors obtained from simulation replica k into

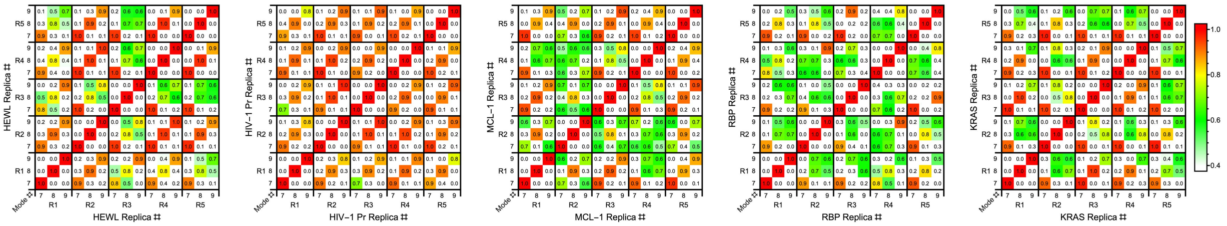

Figure S5: **Zero-frequency mode correlations between all replicas.** Full correlation matrices between all pairs of zero-frequency FRESEAN modes 7-9 obtained for replicas R1 to R5 for each protein system. Colors of individual squares indicate the direct correlation between a pair of modes.

the low-dimensional space defined by eigenvectors obtained from simulation replica l. We then compute the average length of the projected vectors in these low-dimensional projections, which is 1 if the sub-spaces are equivalent, *i.e.*, each eigenvector obtained from simulation replica k can be fully represented as a linear combination of the selected eigenvectors obtained from simulation replica l.

$$C_{\text{2D}}^{(k,l)} = \left(\sum_{i \in 7,8} \sqrt{\sum_{j \in 7,8} \left(Q_i^{(k)} \cdot Q_i^{(l)} \right)^2} \right) / 2 \tag{S13}$$

$$C_{\text{3D}}^{(k,l)} = \left(\sum_{i \in 7,8,9} \sqrt{\sum_{j \in 7,8,9} \left(Q_i^{(k)} \cdot Q_i^{(l)} \right)^2} \right) / 3 \tag{S14}$$

Quasi-harmonic and principal component modes

In contrast to FRESEAN mode analysis, other traditional methods to identify low-frequency vibrations not only fail to isolate low-frequency vibrations due to their reliance on harmonic approximations (see Ref. 36) but also lack reproducibility between distinct simulations. We demonstrate this here for quasi-harmonic normal modes obtained from the same simulation trajectories used for FRESEAN mode analysis.

Quasi-harmonic normal mode analysis is based on the mass-weighted co-variance matrix of atomic displacements relative to an average structure (after alignment with a reference structure). The elements of the displacement co-variance matrix are defined in eq. S15.

$$C_{ij}^{\text{covat}} = \sqrt{m_i \cdot m_j} \cdot \langle (x_i - \langle x_i \rangle) \cdot (x_j - \langle x_j \rangle) \rangle_t \tag{S15}$$

In a harmonic system, the eigenvectors of this matrix correspond to the harmonic normal modes of

the system and the eigenvalues, λ_i, describe the (mass-weighted) variance of projections onto each normal mode. The square-root of the latter is inversely proportional to the harmonic frequency of each normal mode.

$$\omega_i^{\mathrm{QH}} = \sqrt{\frac{k_B T}{\lambda_i}} \tag{S16}$$

Since the co-variance matrix only describes intramolecular distortions, none of the eigenvectors describe translational or rotational motion. Thus we perform the correlation analysis described in the previous section for modes 1-3, *i.e.*, the lowest frequency vibrational modes obtained from quasi-harmonic normal mode analysis. In figure S6, we show the correlations between quasi-harmonic normal modes obtained from replica R1 to the five simulation replicas R1 to R5 for each system, in analogy to the corresponding figure 1 in the main text.

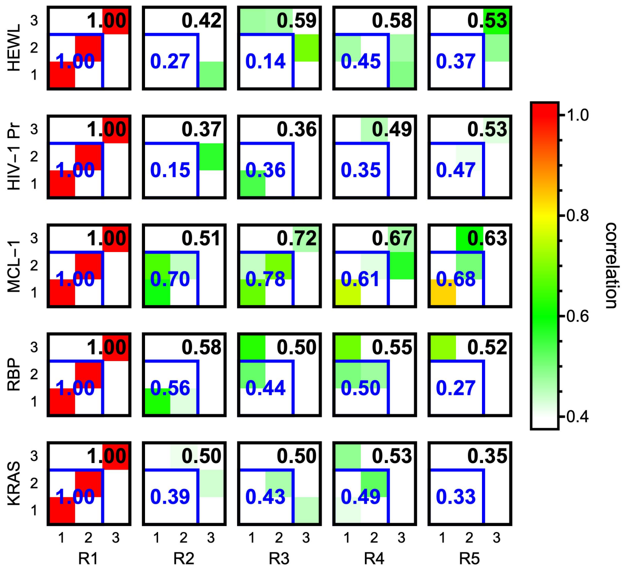

Figure S6: **Reproducibility of quasi-harmonic normal modes in five replica simulations.** Matrices describing correlations between quasi-harmonic normal modes 1-3 in replicas R1 and R1 to R5 for each protein system (self-correlations with replica 1 are 1.0 by definition and only shown for clarity). Colors of individual squares indicate the direct correlation between a pair of modes. Correlation coefficients between 2D (3D) sub-spaces described by modes 1-2 (1-3) in a pair of simulations are shown as blue (black) numerals.

It is apparent that the pair-wise corerlations between modes as well as the correlations between 2D and 3D sub-spaces are drastically reduced compared to low-frequency modes obtained from

FRESEAN mode analysis. The correlations between pairs of quasi-harmonic modes rarely reach values >0.5 and even the correlations between 2D and 3D sub-spaces, which allow for more flexibility, *e.g.*, mixing modes or changing their order, are not improving this picture. Thus, it is clear that FRESEAN mode analysis is substantially more stable and depends less on rare events. For completeness, we also show the correlations between all combinations of simulation replicas in figure S7 in analogy to figure S5 for the FRESEAN modes.

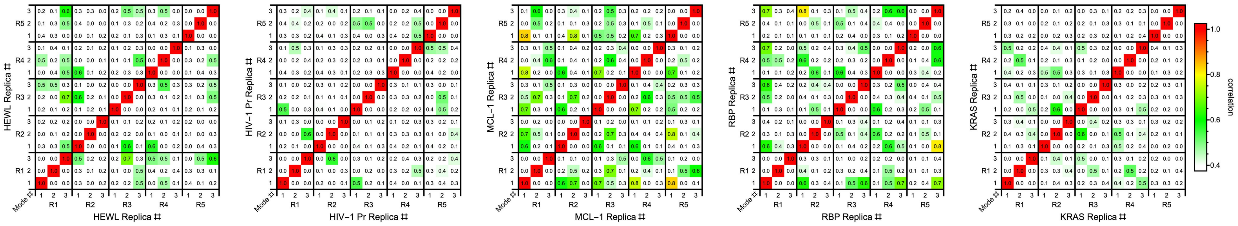

Figure S7: **Quasi-harmonic normal mode correlations between all replicas.** Full correlation matrices between all pairs of quasi-harmonic normal modes 1-3 obtained for replicas R1 to R5 for each protein system. Colors of individual squares indicate the direct correlation between a pair of modes.

To further emphasize the difference between FRESEAN mode analysis and methods based on co-variances of atomic displacements, we selected one system, HEWL, to increase the sampling time per simulation replica from 20 ns to 1000 ns, *i.e.*, 1 microsecond. Applying the same formalism as for quasi-harmonic normal mode analysis, we now obtain principal component modes. The distinction is that for long simulations, the assumption that the system explores a local potential energy minimum is no longer applied. This principal component analysis is useful to identify collective degrees of freedom involved in large conformational changes sampled in the simulation trajectories. The eigenvalues of the co-variance matrix are now directly interpreted as variances of projections along each eigenvector.

We show the pair-wise correlations of principal component modes 1-3 in simulation replica R1 with replica R1 to R5 (all simulated for 1 microsecond) as well as the correlations between 2D and 3D sub-spaces in figure S8. We observe improvements compared to the quasi-harmonic mode analysis, which indicates relatively well-defined conformational dynamics in this system. However, the correlations between the extracted modes are still substantially smaller than for the FRESEAN modes shown in figure 1 in the main text despite a 50-fold increase in the simulation time. As

before, we show the complete set of correlations between all simulation replicas in figure S9.

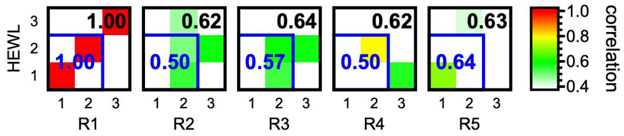

Figure S8: **Reproducibility of principal component modes in five extended replica simulations.** Matrices describing correlations between principal component modes 1-3 in replicas R1 and R1 to R5 (1 microsecond simulations each) for HEWL (self-correlations with replica 1 are 1.0 by definition and only shown for clarity). Colors of individual squares indicate the direct correlation between a pair of modes. Correlation coefficients between 2D (3D) sub-spaces described by modes 1-2 (1-3) in a pair of simulations are shown as blue (black) numerals.

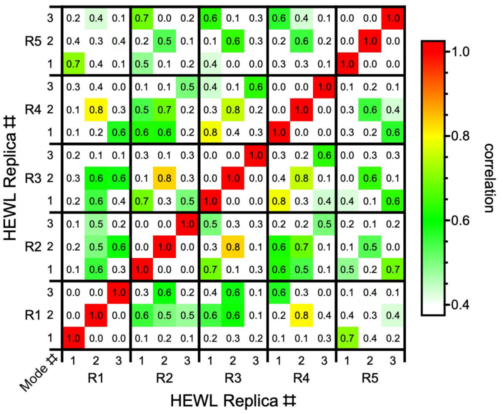

Figure S9: **Principal component mode correlations between all replicas.** Full correlation matrices describing the correlation between all pairs of principal component modes 1-3 between replicas R1 to R5 for HEWL. Colors of individual squares indicate the direct correlation between a pair of modes.

Definition and visualization of geometric variables

As described in the main text, we use geometric variables introduced previously in the literature for the five simulated proteins to analyze the performance of our enhanced sampling scheme. The exact definitions of these collective variables are provided in table S2. We further provide a visualization

of all distances and angles in the context of the respective protein structures in figure 3 of the main text.

Protein	Geometric Variable	Definition/Notes
HEWL	pincer angle [a]	angle between C_α-atom centers of 3 residue groups:
		1. [28-31,111-114]
		2. [90-93]
		3. [44-45,51-52]
	radius of gyration	radius of gyration computed with all protein atoms
HIV-1 Pr	flap distance [a]	distance between C_α-atoms of G51 in each monomer
	flap RMSD	RMSD of residues I50-G52 in both monomers
MCL-1	S255-T226 [b]	distance between C_α-atoms of residues S225 and T226
	interdomain angle [a]	angle between C_α-atoms of residues S225, D241, T226
RBP	twist angle [b]	dihedral angle between COMs of 4 groups of residues:
		1. [1-100,236-259]
		2. [99-100,236-237,258-259]
		3. [108-109,230-231,269-270],
		4. [108-231,269-271]
	hinge angle [a]	angle between COMs of 3 groups of residues:
		1. [1-100,236-259]
		2. [101-107,232-235,260-268]
		3. [108-231,269-271]
KRAS	G12-T35 [b]	distance between C_α atoms of residues G12 and T35
	T35-G60 [a]	distance between C_α atoms of residues T35 and G60

[a] shown in magenta in figure 3A; [b] shown in cyan in figure 3A

Table S2: Geometric Variables for Analysis

”Closed” and ”open” conformations

In the main text, we use the terms ”closed” and ”open” as a common denomination for previously observed conformations of each protein. The corresponding structures are shown in blue (closed)

and red (open) in figure 3 of the main text. These states are also indicated as blue and red symbols in figures 4-6 of the main text as well as in figures S10, S12, and S13 below.

Extended simulations

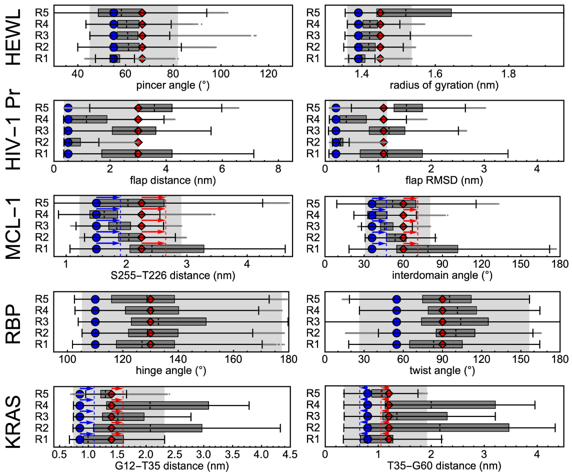

Figure S10: **Extended metadynamics simulations.** Box and whisker plots for metadynamics simulations as shown in Figure 4 of the main text after extending the trajectories to 160 ns length. We observe consistent sampling of conformational transitions between the “closed” and “open” states for all five replicas of all five systems. Individual replicas of some systems, *e.g.*, R5 of HEWL, begin to explore non-native conformations at high free energies and thus does not generate new information on the folded state ensemble.

Averaged free energy surfaces

To obtain the converged free energy surfaces shown in figure 5 of the main text, we ran unbiased 100 ns NPT simulations for each protein and extract from it 20 configurations (every 5 ns). These are then used as starting points for 20 metadynamics simulations using the zero frequency modes 7 and 8 obtained from FRESEAN mode analysis for replica R1 for each system, while otherwise using the same metadynamics simulation protocol as described for replicas R1 to R5. To obtain averaged free energy surfaces from multiple metadynamics simulations, we first compute for each

simulation an unbiased probability distribution as a function of its CVs, *e.g.*, CV_1 and CV_2.

$$p(CV_1, CV_2) = \frac{1}{\Omega} \exp\left[\frac{E_{\text{bias}}(CV_1, CV_2)}{k_B T}\right] \tag{S17}$$

Here, E_{bias} is the biasing potential generated as a sum of Gaussians during metadynamics sampling (describes a negative free energy) and Ω is the corresponding partition function, which here serves simply as a normalization constant.

$$\Omega = \int_{-\infty}^{+\infty} \int_{-\infty}^{+\infty} \exp\left[\frac{E_{\text{bias}}(CV_1, CV_2)}{k_B T}\right] dCV_1\, dCV_2 \tag{S18}$$

We then compute the average probability distribution $\langle p(CV_1, CV_2)\rangle$ over the 20 simulations before converting into a free energy surface.

$$\langle p(CV_1, CV_2)\rangle = \sum_{i=1}^{20} p_i(CV_1, CV_2)/20 \tag{S19}$$

$$\Delta G(CV_1, CV_2) = -k_B T \ln\left[\langle p(CV_1, CV_2)\rangle\right] \tag{S20}$$

Free energy surfaces in distinct variable spaces

To define free energy surfaces as a function of geometric variables, *e.g.*, as described in table S2, using biased simulation trajectories generated with bias potentials applied to a distinct set of CVs, *e.g.*, low-frequency vibrations, we generate a weighted ensemble of structures based on the probabilities given in eq. S17. For each time frame t_i of the trajectory, we compute the CVs used to define E_{bias} and evaluate $p(CV_1, CV_2)$ to obtain its relative weight.

$$w(t_i) = p\left[CV_1(t_i), CV_2(t_i)\right] \tag{S21}$$

We then compute the unbiased probability distribution for any other set of variables, *e.g.*, h_1 and h_2 that can be expressed in terms of the system coordinates at time t_i.

$$p(h_1, h_2) = \frac{\sum_i^{n_t} w(t_i) \cdot \delta\left[h_1'(t_i) - h_1\right] \cdot \delta\left[h_2'(t_i) - h_2\right]}{\sum_i^{n_t} w(t_i)} \tag{S22}$$

Here, δ indicates the Kronecker delta function with $\delta[0] = 1$ and $\delta[x \neq 0] = 0$. In practice, the delta function is replaced by a histogram bin of finite size. The probability function $p(h_1, h_2)$ now describes a weighted ensemble as a function of the variables h_1 and h_2 for which we can define the free energy surface.

$$\Delta G(h_1, h_2) = -k_B T \ln\left[p(h_1, h_2)\right] \tag{S23}$$

Box & whisker plot definition

The box-and-whisker plots shown in figure 4 of the main text and figure S10 are defined as follows. The dark gray box represents the inter-quartile range, which is defined from the 25th percentile to the 75th percentile. The dashed vertical line denotes the median value of the dataset. The lower whisker position is defined as the dataset minimum or 1.5x the inter-quartile range, whichever is smaller. The higher whisker position is determined in a similar way, except with the 75th percentile and the dataset maximum. Outliers are defined as points outside of the whisker range.

Unbiased control simulations

To verify that our metadynamics simulations with low-frequency vibrational modes as CVs indeed achieve enhanced sampling, we performed unbiased control simulations with otherwise identical simulation parameters (five replicas R1 to R5 for each system with 100 ns sampling time).

Free energy surfaces for individual replicas in CV-space

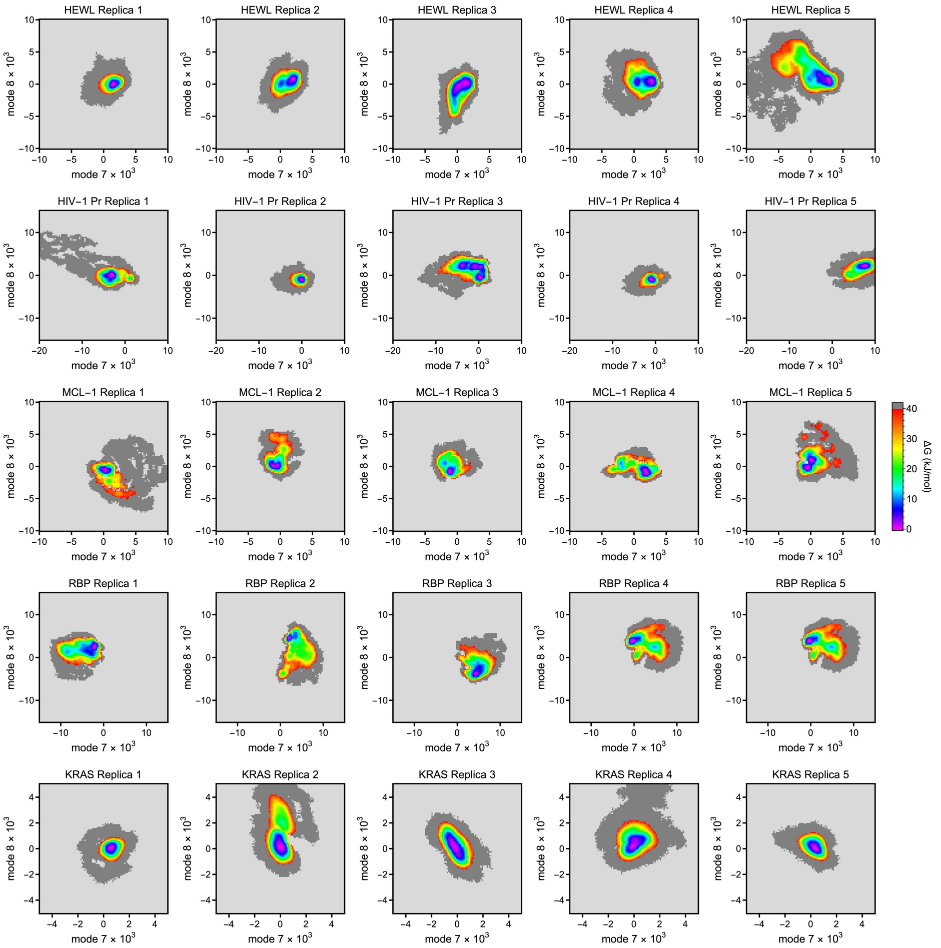

Figure S11: **Individual replica simulations in CV-space.** FESs of replicas R1 to R5 for each protein system obtained from 100 ns metadynamics simulations as a function of vibrational modes used as CVs. The specific CVs for each replica were obtained from independent 20 ns simulations using FRESEAN mode analysis and are thus not identical despite the high correlations shown in figure 1 of the main text. Even trivial differences that have no impact on enhanced sampling can significantly alter the appearance of each replica FES, which is why a direct comparison is not possible. For example, switches in sign of the corresponding eigenvectors have no impact on enhanced sampling or the correlation coefficient defined in eq. S12, but the corresponding FES would be inverted. Likewise, distinct linear combinations of the two selected eigenvectors have no impact on enhanced sampling or correlations of the two-dimensional sub-space defined by them, but the FES would be rotated.

Free energy surfaces for individual replicas in geometric space

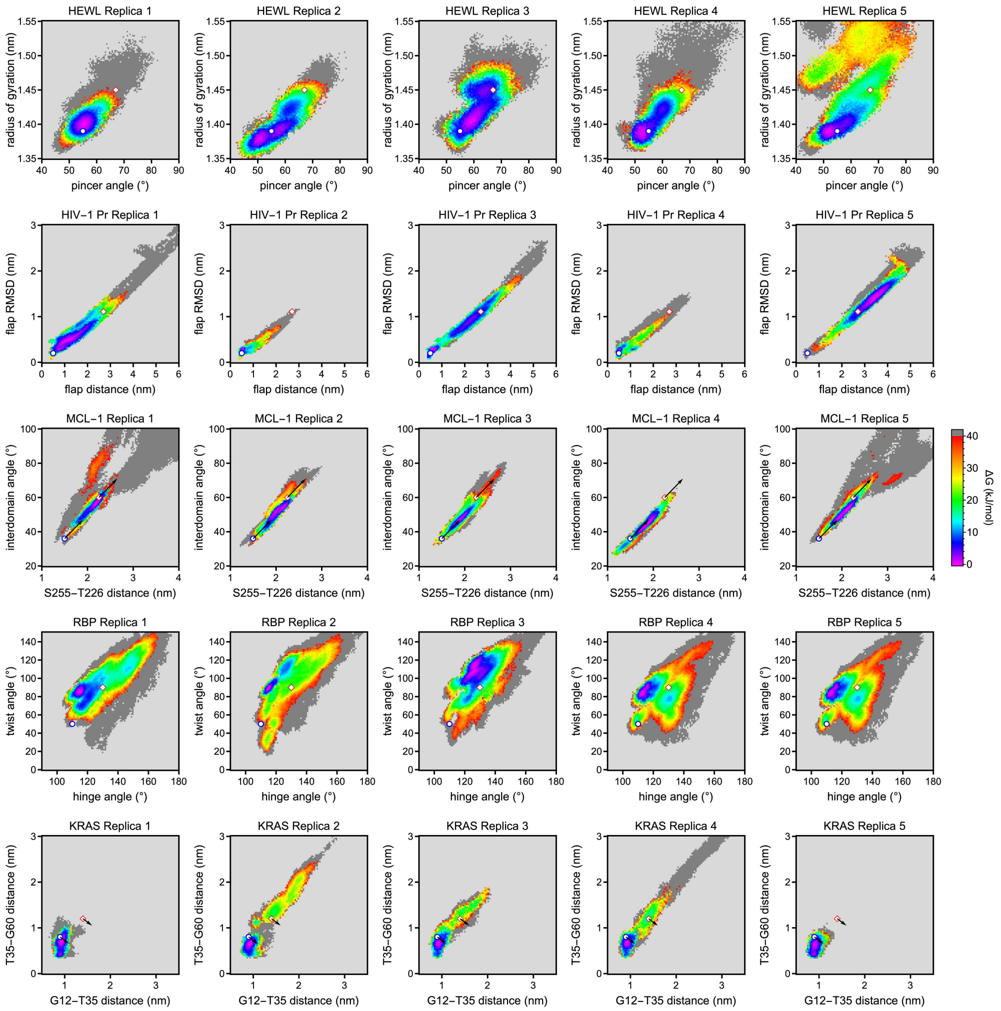

Figure S12: **Individual replica simulations reweighted.** FESs of replicas R1 to R5 for each protein system obtained from 100 ns metadynamics simulations as a function of geometric variables defined in table S2 and figure 3A. These FESs were obtained from weighted ensembles as described in Eqs. S21 to S23 and can be compared between the five replicas for each system despite the use of distinct CVs during metadynamics (see caption of figure S11). While common themes are apparent for different replicas for each system, we performed a second set of 20 replica metadynamics simulations (using identical CVs) to obtain the converged free energy surfaces shown in figure 5 of the main text.

Minimum free energy path for conformational transition in HEWL

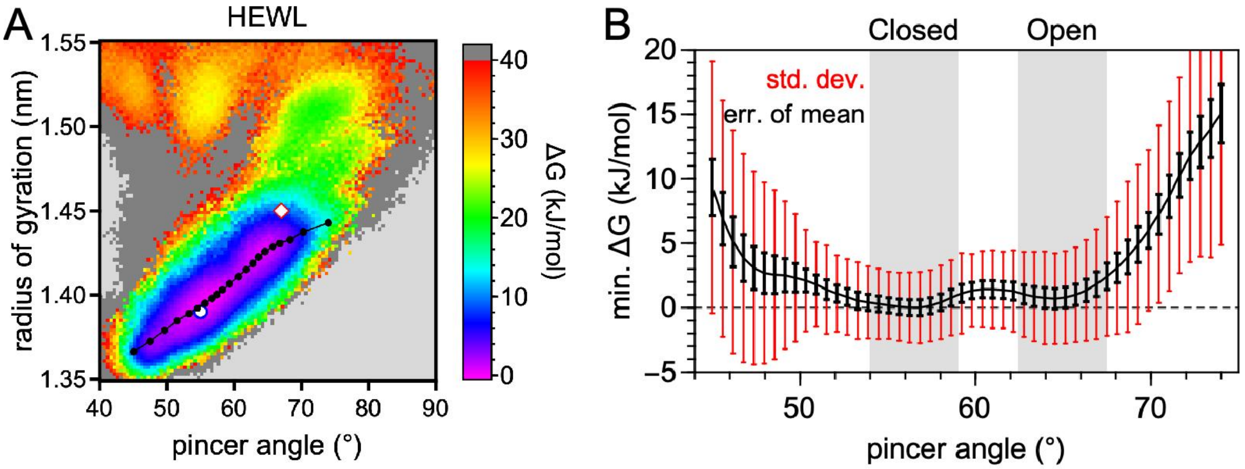

Figure S13: **Minimum free energy path for HEWL.** To further visualize the statistical convergence of free energy surfaces obtained from 20 independent metadynamics simulations using low-frequency modes obtained from FRESEAN mode analysis as CVs, we re-plot in panel A the converged free energy surface for HEWL as a function of its geometric variables (same as in figure 5 of the main text), while indicating the minimum free energy pathway connecting the "closed" and "open" conformations (dashed line). In panel B, we plot the average free energy profile along the minimum free energy path as a function of the pincer angle. Gray boxes are centered on the location of "closed" and "open" states as reported in the literature. These do not exactly correspond to the minima of our free energy surface, but fall within larger regions in which we find the FES to be approximately flat. In addition, we show error bars representing the standard deviation (red; uncertainty of a single 100 ns simulation) and the error of the mean obtained after averaging over 20 simulations.

Free energy differences between ”closed” and ”open” states

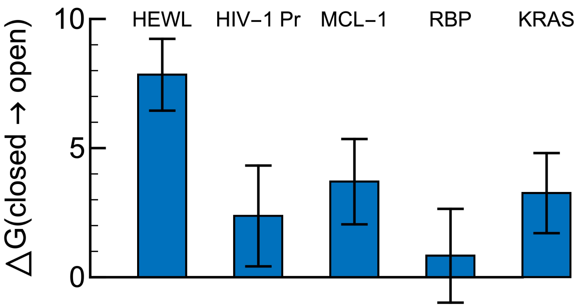

Figure S14: **Free energy differences.** Free energy differences and statistical uncertainties between ”closed” and ”open” states for all five systems as described by the corresponding free energy minima (including reported shifts for MCL-1 and KRAS) in figure 5B.

Partial unfolding of KRAS upon biasing along residue-residue distances

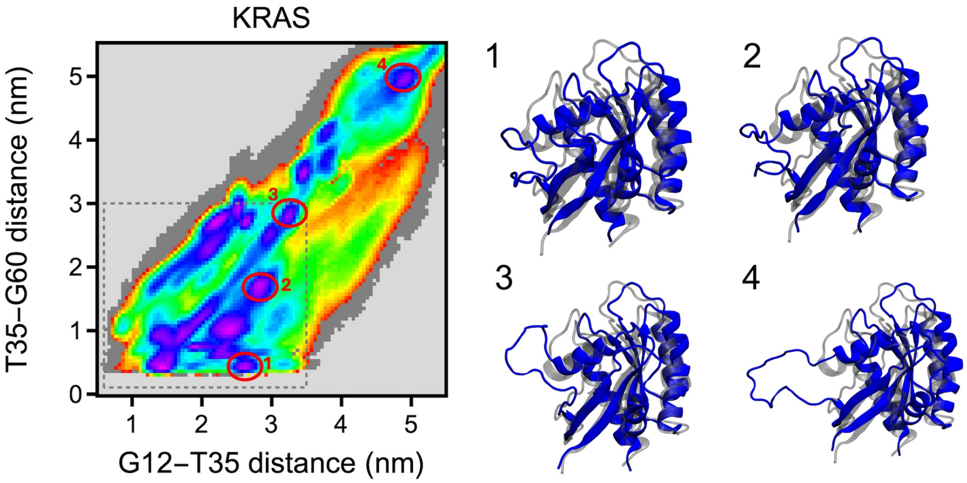

Figure S15: **Partial unfolding of KRAS.** WT-metadynamics simulations with biasing potentials applied directly to residue-residue distances (see table S2) frequently explore unfolded conformations. Left: Average FES with an extended plot range (dashed box indicates plot range in figure 6A). Specific free energy minima are highlighted with red numbers and ellipses. Right: Visualization of structures corresponding to the highlighted free energy minima indicated in the left panel (blue) with a folded reference structure (gray).

Shannon entropy

To determine the information content of an FES expressed in a given two-dimensional CV-space, we first convert it into the corresponding probability distribution as defined in eq. S17. Deviating from our notation elsewhere, we acknowledge the discretized nature of probability distributions generated as histograms from numerical simulations. The information content can be quantified in terms of the Shannon entropy, which we express here in units of bits:

$$S_2 = -\sum_i \sum_j p\left(CV_{1,i}, CV_{2,j}\right) \log_2 \left[p\left(CV_{1,i}, CV_{2,j}\right)\right] \tag{S24}$$

Bhattacharyya coefficient

To quantify the similarity of a pair of FES expressed in the same two-dimensional CV-space, we express both in terms of their corresponding probability distributions p_A and p_B as described in eq. S17. The Bhattacharyya coefficient (BC) describes their overlap via the integral:

$$\mathrm{BC} = \int_{-\infty}^{+\infty} \int_{-\infty}^{+\infty} \sqrt{p_A\left(CV_1, CV_2\right) \cdot p_B\left(CV_1, CV_2\right)}\ dCV_1 dCV_2 \tag{S25}$$

REFERENCES

1. A. W. Senior, R. Evans, J. Jumper, J. Kirkpatrick, L. Sifre, T. Green, C. Qin, A. Žídek, A. W. R. Nelson, A. Bridgland, H. Penedones, S. Petersen, K. Simonyan, S. Crossan, P. Kohli, D. T. Jones, D. Silver, K. Kavukcuoglu, D. Hassabis, Improved protein structure prediction using potentials from deep learning. *Nature* **577**, 706–710 (2020).

2. J. Jumper, R. Evans, A. Pritzel, T. Green, M. Figurnov, O. Ronneberger, K. Tunyasuvunakool, R. Bates, A. Žídek, A. Potapenko, A. Bridgland, C. Meyer, S. A. A. Kohl, A. J. Ballard, A. Cowie, B. Romera-Paredes, S. Nikolov, R. Jain, J. Adler, T. Back, S. Petersen, D. Reiman, E. Clancy, M. Zielinski, M. Steinegger, M. Pacholska, T. Berghammer, S. Bodenstein, D. Silver, O. Vinyals, A. W. Senior, K. Kavukcuoglu, P. Kohli, D. Hassabis, Highly accurate protein structure prediction with AlphaFold. *Nature* **596**, 583–589 (2021).

3. M. Gao, D. Nakajima An, J. M. Parks, J. Skolnick, AF2Complex predicts direct physical interactions in multimeric proteins with deep learning. *Nat. Commun.* **13**, 1744 (2022).

4. J. Abramson, J. Adler, J. Dunger, R. Evans, T. Green, A. Pritzel, O. Ronneberger, L. Willmore, A. J. Ballard, J. Bambrick, S. W. Bodenstein, D. A. Evans, C.-C. Hung, M. O'Neil, D. Reiman, K. Tunyasuvunakool, Z. Wu, A. Žemgulytė, E. Arvaniti, C. Beattie, O. Bertolli, A. Bridgland, A. Cherepanov, M. Congreve, A. I. Cowen-Rivers, A. Cowie, M. Figurnov, F. B. Fuchs, H. Gladman, R. Jain, Y. A. Khan, C. M. R. Low, K. Perlin, A. Potapenko, P. Savy, S. Singh, A. Stecula, A. Thillaisundaram, C. Tong, S. Yakneen, E. D. Zhong, M. Zielinski, A. Žídek, V. Bapst, P. Kohli, M. Jaderberg, D. Hassabis, J. M. Jumper, Accurate structure prediction of biomolecular interactions with AlphaFold 3. *Nature* **630**, 493–500 (2024).

5. Z. Lin, H. Akin, R. Rao, B. Hie, Z. Zhu, W. Lu, N. Smetanin, R. Verkuil, O. Kabeli, Y. Shmueli, A. dos Santos Costa, M. Fazel-Zarandi, T. Sercu, S. Candido, A. Rives, Evolutionary-scale prediction of atomic-level protein structure with a language model. *Science* **379**, 1123–1130 (2023).

6. M. Baek, F. DiMaio, I. Anishchenko, J. Dauparas, S. Ovchinnikov, G. R. Lee, J. Wang, Q. Cong, L. N. Kinch, R. D. Schaeffer, C. Millán, H. Park, C. Adams, C. R. Glassman, A. DeGiovanni, J. H. Pereira, A. V. Rodrigues, A. van Dijk, A. C. Ebrecht, D. J. Opperman, T.

Sagmeister, C. Buhlheller, T. Pavkov-Keller, M. K. Rathinaswamy, U. Dalwadi, C. K. Yip, J. E. Burke, K. C. Garcia, N. V. Grishin, P. D. Adams, R. J. Read, D. Baker, Accurate prediction of protein structures and interactions using a three-track neural network. *Science* **373**, 871–876 (2021).

7. R. Krishna, J. Wang, W. Ahern, P. Sturmfels, P. Venkatesh, I. Kalvet, G. R. Lee, F. S. Morey-Burrows, I. Anishchenko, I. R. Humphreys, R. McHugh, D. Vafeados, X. Li, G. A. Sutherland, A. Hitchcock, C. N. Hunter, A. Kang, E. Brackenbrough, A. K. Bera, M. Baek, F. DiMaio, D. Baker, Generalized biomolecular modeling and design with RoseTTAFold All-Atom. *Science* **384**, eadl2528 (2024).

8. J. L. Watson, D. Juergens, N. R. Bennett, B. L. Trippe, J. Yim, H. E. Eisenach, W. Ahern, A. J. Borst, R. J. Ragotte, L. F. Milles, B. I. M. Wicky, N. Hanikel, S. J. Pellock, A. Courbet, W. Sheffler, J. Wang, P. Venkatesh, I. Sappington, S. V. Torres, A. Lauko, V. De Bortoli, E. Mathieu, S. Ovchinnikov, R. Barzilay, T. S. Jaakkola, F. D. Maio, M. Baek, D. Baker, De novo design of protein structure and function with RFdiffusion. *Nature* **620**, 1089–1100 (2023).

9. D. Petrović, V. A. Risso, S. C. L. Kamerlin, J. M. Sanchez-Ruiz, Conformational dynamics and enzyme evolution. *J. Roy. Soc. Interface* **15**, 20180330 (2018).

10. P. Campitelli, T. Modi, S. Kumar, S. B. Ozkan, The role of conformational dynamics and allostery in modulating protein evolution. *Annu. Rev. Biophys.* **49**, 267–288 (2020).

11. P. Ayaz, A. Lyczek, Y. T. Paung, V. R. Mingione, R. E. Iacob, P. W. de Waal, J. R. Engen, M. A. Seeliger, Y. Shan, D. E. Shaw, Structural mechanism of a drug-binding process involving a large conformational change of the protein target. *Nat. Commun.* **14**, 1885 (2023).

12. S. Lewis, T. Hempel, J. Jiménez-Luna, M. Gastegger, Y. Xie, A. Y. K. Foong, V. G. Satorras, O. Abdin, B. S. Veeling, I. Zaporozhets, Y. Chen, S. Yang, A. E. Foster, A. Schneuing, J. Nigam, F. Barbero, V. Stimper, A. Campbell, J. Yim, M. Lienen, Y. Shi, S. Zheng, H. Schulz, U. Munir, R. Sordillo, R. Tomioka, C. Clementi, F. Noé, Scalable emulation of protein equilibrium ensembles with generative deep learning. *Science* **389**, eadv9817 (2025).

13. H. M. Berman, J. Westbrook, Z. Feng, G. Gilliland, T. N. Bhat, H. Weissig, I. N. Shindyalov, P. E. Bourne, The protein data bank. *Nucleic Acids Res.* **28**, 235–242 (2000).

14. G. Brändén, R. Neutze, Advances and challenges in time-resolved macromolecular crystallography. *Science* **373**, eaba0954 (2021).

15. D. R. Hekstra, Emerging time-resolved x-ray diffraction approaches for protein dynamics. *Annu. Rev. Biophys.* **52**, 255–274 (2023).

16. N. Fischer, A. L. Konevega, W. Wintermeyer, M. V. Rodnina, H. Stark, Ribosome dynamics and tRNA movement by time-resolved electron cryomicroscopy. *Nature* **466**, 329–333 (2010).

17. J. Zhao, S. Benlekbir, J. L. Rubinstein, Electron cryomicroscopy observation of rotational states in a eukaryotic V-ATPase. *Nature* **521**, 241–245 (2015).

18. E. J. Rundlet, M. Holm, M. Schacherl, S. K. Natchiar, R. B. Altman, C. M. T. Spahn, A. G. Myasnikov, S. C. Blanchard, Structural basis of early translocation events on the ribosome. *Nature* **595**, 741–745 (2021).

19. D. Nettels, N. Galvanetto, M. T. Ivanović, M. Nüesch, T. Yang, B. Schuler, Single-molecule FRET for probing nanoscale biomolecular dynamics. *Nat. Rev. Phys.* **6**, 587–605 (2024).

20. S. Hohng, S. Lee, J. Lee, M. H. Jo, Maximizing information content of single-molecule FRET experiments: Multi-color FRET and FRET combined with force or torque. *Chem. Soc. Rev.* **43**, 1007–1013 (2014).

21. M. Götz, P. Wortmann, S. Schmid, T. Hugel, A multicolor single-molecule FRET approach to study protein dynamics and interactions simultaneously. *Methods Enzymol.* **581**, 487–516 (2016).

22. H. S. Chung, W. A. Eaton, Protein folding transition path times from single molecule FRET. *Curr. Opin. Struct. Biol.* **48**, 30–39 (2018).

23. J. Yoo, J.-Y. Kim, J. M. Louis, I. V. Gopich, H. S. Chung, Fast three-color single-molecule FRET using statistical inference. *Nat. Commun.* **11**, 3336 (2020).

24. A. Barducci, G. Bussi, M. Parrinello, Well-tempered metadynamics: A smoothly converging and tunable free-energy method. *Phys. Rev. Lett.* **100**, 020603 (2008).

25. M. C. Zwier, J. L. Adelman, J. W. Kaus, A. J. Pratt, K. F. Wong, N. B. Rego, E. Suárez, S. Lettieri, D. W. Wang, M. Grabe, D. M. Zuckerman, L. T. Chong, WESTPA: An interoperable, highly scalable software package for weighted ensemble simulation and analysis. *J. Chem. Theory Comput.* **11**, 800–809 (2015).

26. K. Lindorff-Larsen, S. Piana, R. O. Dror, D. E. Shaw, How fast-folding proteins fold. *Science* **334**, 517–520 (2011).

27. K. Lindorff-Larsen, P. Maragakis, S. Piana, D. E. Shaw, Picosecond to millisecond structural dynamics in human ubiquitin. *J. Phys. Chem. B.* **120**, 8313–8320 (2016).

28. D. E. Shaw, P. J. Adams, A. Azaria, J. A. Bank, B. Batson, A. Bell, M. Bergdorf, J. Bhatt, J. Adam Butts, T. Correia, R. M. Dirks, R. O. Dror, M. P. Eastwood, B. Edwards, A. Even, P. Feldmann, M. Fenn, C. H. Fenton, A. Forte, J. Gagliardo, G. Gill, M. Gorlatova, B. Greskamp, J. P. Grossman, J. Gullingsrud, A. Harper, W. Hasenplaugh, M. Heily, B. C. Heshmat, J. Hunt, D. J. Ierardi, L. Iserovich, B. L. Jackson, N. P. Johnson, M. M. Kirk, J. L. Klepeis, J. S. Kuskin, K. M. Mackenzie, R. J. Mader, R. M. Gowen, A. M. Laughlin, M. A. Moraes, M. H. Nasr, L. J. Nociolo, L. O'Donnell, A. Parker, J. L. Peticolas, G. Pocina, C. Predescu, T. Quan, J. K. Salmon, C. Schwink, K. S. Shim, N. Siddique, J. Spengler, T. Szalay, R. Tabladillo, R. Tartler, A. G. Taube, M. Theobald, B. Towles, W. Vick, S. C. Wang, M. Wazlowski, M. J. Weingarten, J. M. Williams, K. A. Yuh, "Anton 3: Twenty microseconds of molecular dynamics simulation before lunch," in *Proceedings of the International Conference for High Performance Computing, Networking, Storage and Analysis* (IEEE, 2021), pp. 1–11; DOI: 10.1145/3458817.3487397.

29. M. Zimmerman, J. R. Porter, M. D. Ward, S. Singh, N. Vithani, A. Meller, U. L. Mallimadugula, C. E. Kuhn, J. H. Borowsky, R. P. Wiewiora, M. F. D. Hurley, A. M. Harbison, C. A. Fogarty, J. E. Coffland, E. Fadda, V. A. Voelz, J. D. Chodera, G. R. Bowman, SARS-CoV-2 simulations go exascale to predict dramatic spike opening and cryptic pockets across the proteome. *Nat. Chem.* **13**, 651–659 (2021).

30. Z. F. Brotzakis, M. Parrinello, Enhanced sampling of protein conformational transitions via dynamically optimized collective variables. *J. Chem. Theory Comput.* **15**, 1393–1398 (2019).

31. L. Bonati, E. Trizio, A. Rizzi, M. Parrinello, A unified framework for machine learning collective variables for enhanced sampling simulations: MLCOLVAR. *J. Chem. Phys.* **159**, 014801 (2023).

32. K. Shmilovich, A. L. Ferguson, Girsanov reweighting enhanced sampling technique (grest): On-the-fly data-driven discovery of and enhanced sampling in slow collective variables. *J. Phys. Chem. A* **127**, 3497–3517 (2023).

33. J. Rydzewski, M. Chen, T. K. Ghosh, O. Valsson, Reweighted manifold learning of collective variables from enhanced sampling simulations. *J. Chem. Theory Comput.* **18**, 7179–7192 (2022).

34. S. Mehdi, Z. Smith, L. Herron, Z. Zou, P. Tiwary, Enhanced sampling with machine learning. *Ann. Rev. Phys. Chem.* **75**, 347–370 (2024).

35. W. Shen, K. Wan, D. Li, H. Gao, X. Shi, Adaptive CVgen: Leveraging reinforcement learning for advanced sampling in protein folding and chemical reactions. *Proc. Natl. Acad. Sci. U.S.A.* **121**, e2414205121 (2024).

36. M. A. Sauer, M. Heyden, Frequency-selective anharmonic mode analysis of thermally excited vibrations in proteins. *J. Chem. Theory Comput.* **19**, 5481–5490 (2023).

37. B. Brooks, M. Karplus, Harmonic dynamics of proteins: Normal modes and fluctuations in bovine pancreatic trypsin inhibitor. *Proc. Natl. Acad. Sci. U.S.A.* **80**, 6571–6575 (1983).

38. L. Yang, G. Song, R. L. Jernigan, How well can we understand large-scale protein motions using normal modes of elastic network models? *Biophys. J.* **93**, 920–929 (2007).

39. S. Mahajan, Y.-H. Sanejouand, Jumping between protein conformers using normal modes. *J. Comput. Chem.* **38**, 1622–1630 (2017).

40. M. G. Costa, P. R. Batista, A. Gomes, L. S. Bastos, M. Louet, N. Floquet, P. M. Bisch, D. Perahia, MDexciteR: Enhanced sampling molecular dynamics by excited normal modes or principal components obtained from experiments. *J. Chem. Theory Comput.* **19**, 412–425 (2023).

41. S. Mondal, M. A. Sauer, M. Heyden, Exploring conformational landscapes along anharmonic low-frequency vibrations. *J. Phys. Chem. B* **128**, 7112–7120 (2024).

42. Y. M. Huang, J. A. McCammon, Y. Miao, Replica exchange Gaussian accelerated molecular dynamics: Improved enhanced sampling and free energy calculation. *J. Chem. Theory Comput.* **14**, 1853–1864 (2018).

43. M. Benabderrahmane, R. Bureau, A. S. Voisin-Chiret, J. Sopkova-de Oliveira Santos, Insights into Mcl-1 conformational states and allosteric inhibition mechanism from molecular dynamics simulations, enhanced sampling, and pocket crosstalk analysis. *J. Chem. Inf. Model.* **60**, 3172–3187 (2020).

44. W. Ren, H. M. Dokainish, A. Shinobu, H. Oshima, Y. Sugita, Unraveling the coupling between conformational changes and ligand binding in ribose binding protein using multiscale molecular dynamics and free-energy calculations. *J. Phys. Chem. B* **125**, 2898–2909 (2021).

45. J. Chen, S. Zhang, W. Wang, L. Pang, Q. Zhang, X. Liu, Mutation-induced impacts on the switch transformations of the GDP- and GTP-bound K-ras: Insights from multiple replica Gaussian accelerated molecular dynamics and free energy analysis. *J. Chem. Inf. Model.* **61**, 1954–1969 (2021).

46. M. A. Sauer, S. Mondal, M. Cano, M. Heyden, High-throughput computation of anharmonic low-frequency protein vibrations. *J. Phys. Chem. B* **129**, 10739–10751 (2025).

47. A. De Simone, R. W. Montalvao, C. M. Dobson, M. Vendruscolo, Characterization of the interdomain motions in hen lysozyme using residual dipolar couplings as replica-averaged structural restraints in molecular dynamics simulations. *Biochemistry* **52**, 6480–6486 (2013).

48. D. Branduardi, G. Bussi, M. Parrinello, Metadynamics with adaptive Gaussians. *J. Chem. Theory Comput.* **8**, 2247–2254 (2012).

49. J. F. Dama, G. M. Hocky, R. Sun, G. A. Voth, Exploring valleys without climbing every peak: More efficient and forgiving metabasin metadynamics via robust on-the-fly bias domain restriction. *J. Chem. Theory Comput.* **11**, 5638–5650 (2015).

50. M. Levitt, C. Sander, P. S. Stern, Protein normal-mode dynamics: Trypsin inhibitor, crambin, ribonuclease and lysozyme. *J. Mol. Biol.* **181**, 423–447 (1985).

51. C. Chennubhotla, A. Rader, L.-W. Yang, I. Bahar, Elastic network models for understanding biomolecular machinery: From enzymes to supramolecular assemblies. *Phys. Biol.* **2**, S173–S180 (2005).

52. UniProt Consortium, UniProt: A worldwide hub of protein knowledge. *Nucleic Acids Res.* **47**, D506–D515 (2019).

53. G. Janson, G. Valdes-Garcia, L. Heo, M. Feig, Direct generation of protein conformational ensembles via machine learning. *Nat. Commun.* **14**, 774 (2023).

54. G. Tesei, A. I. Trolle, N. Jonsson, J. Betz, F. E. Knudsen, F. Pesce, K. E. Johansson, K. Lindorff-Larsen, Conformational ensembles of the human intrinsically disordered proteome. *Nature* **626**, 897–904 (2024).

55. J. M. Lotthammer, G. M. Ginell, D. Griffith, R. Emenecker, A. S. Holehouse, Direct prediction of intrinsically disordered protein conformational properties from sequence. *Nat. Methods* **21**, 465–476 (2024).

56. D. M. Jennewein, J. Lee, C. Kurtz, W. Dizon, I. Shaeffer, A. Chapman, A. Chiquete, J. Burks, A. Carlson, N. Mason, A. Kobawala, T. Jagadeesan, P. B. Basani, T. Battelle, R. Belshe, D. M. Caffrey, M. Brazil, C. Inumella, K. Kuznia, J. Buzinski, D. D. Shah, S. M. Dudley, G. Speyer, J. Yalim, "The Sol supercomputer at Arizona State University," in *Practice and Experience in Advanced Research Computing, PEARC '23* (Association for Computing Machinery, 2023), pp. 296–301; DOI:10.1145/3569951.3597573.

57. M. A. Sauer, S. Mondal, M. Heyden, FRESEAN-metadynamics (2025); https://github.com/HeydenLabASU-collab/FRESEAN-metadynamics.

58. M. J. Abraham, T. Murtola, R. Schulz, S. Páll, J. C. Smith, B. Hess, E. Lindahl, GROMACS: High performance molecular simulations through multi-level parallelism from laptops to supercomputers. *SoftwareX* **1**, 19–25 (2015).

59. W.-L. Jorgensen, J. Chandrasekhar, J.-D. Madura, R.-W. Impey, M.-L. Klein, Comparison of simple potential functions for simulating liquid water. *J. Chem. Phys.* **79**, 926–935 (1983).

60. A. D. MacKerell Jr., D. Bashford, M. Bellott, R. L. Dunbrack Jr., J. D. Evanseck, M. J. Field, S. Fischer, J. Gao, H. Guo, S. Ha, D. J.-M. Carthy, L. Kuchnir, K. Kuczera, F. T. K. Lau, C. Mattos, S. Michnick, T. Ngo, D. T. Nguyen, B. Prodhom, W. E. Reiher, B. Roux, M. Schlenkrich, J. C. Smith, R. Stote, J. Straub, M. Watanabe, J. Wiórkiewicz-Kuczera, D. Yin, M. Karplus, All-atom empirical potential for molecular modeling and dynamics studies of proteins. *J. Phys. Chem. B* **102**, 3586–3616 (1998).

61. V. Hornak, R. Abel, A. Okur, B. Strockbine, A. Roitberg, C. Simmerling, Comparison of multiple Amber force fields and development of improved protein backbone parameters. *Proteins* **65**, 712–725 (2006).

62. J. A. Maier, C. Martinez, K. Kasavajhala, L. Wickstrom, K. E. Hauser, C. Simmerling, ff14SB: Improving the accuracy of protein side chain and backbone parameters from ff99SB. *J. Chem. Theory Comput.* **11**, 3696–3713 (2015).

63. J. Huang, S. Rauscher, G. Nawrocki, T. Ran, M. Feig, B. L. de Groot, H. Grubmüller, A. D. MacKerell Jr., CHARMM36m: An improved force field for folded and intrinsically disordered proteins. *Nat. Methods* **14**, 71–73 (2017).

64. G. Bussi, D. Donadio, M. Parrinello, Canonical sampling through velocity rescaling. *J. Chem. Phys.* **126**, 014101 (2007).

65. M. Bernetti, G. Bussi, Pressure control using stochastic cell rescaling. *J. Chem. Phys.* **153**, 114107 (2020).

66. S. Nosé, A molecular dynamics method for simulations in the canonical ensemble. *Mol. Phys.* **52**, 255–268 (1984).

67. W. G. Hoover, Canonical dynamics: Equilibrium phase-space distributions. *Phys. Rev. A* **31**, 1695–1697 (1985).

68. M. Parrinello, A. Rahman, Polymorphic transitions in single crystals: A new molecular dynamics method. *J. Appl. Phys.* **52**, 7182–7190 (1981).

69. B. Hess, H. Bekker, H. J. Berendsen, J. G. Fraaije, LINCS: A linear constraint solver for molecular simulations. *J. Comput. Chem.* **18**, 1463–1472 (1997).

70. T. Darden, D. York, L. Pedersen, Particle mesh Ewald: An N log (N) method for Ewald sums in large systems. *J. Chem. Phys.* **98**, 10089–10092 (1993).

71. M. Bonomi, D. Branduardi, G. Bussi, C. Camilloni, D. Provasi, P. Raiteri, D. Donadio, F. Marinelli, F. Pietrucci, R. A. Broglia, M. Parrinello, PLUMED: A portable plugin for free-energy calculations with molecular dynamics. *Comput. Phys. Commun.* **180**, 1961–1972 (2009).

72. G. A. Tribello, M. Bonomi, D. Branduardi, C. Camilloni, G. Bussi, PLUMED 2: New feathers for an old bird. *Comput. Phys. Commun.* **185**, 604–613 (2014).
